# A de novo approach to inferring within-host fitness effects during untreated HIV-1 infection

**DOI:** 10.1371/journal.ppat.1008171

**Published:** 2020-06-03

**Authors:** Christopher J. R. Illingworth, Jayna Raghwani, David Serwadda, Nelson K. Sewankambo, Merlin L. Robb, Michael A. Eller, Andrew R. Redd, Thomas C. Quinn, Katrina A. Lythgoe

**Affiliations:** 1 Department of Genetics, University of Cambridge, Cambridge, United Kingdom; 2 Department of Applied Mathematics and Theoretical Physics, University of Cambridge, Cambridge, United Kingdom; 3 School of Chemical and Biological Sciences, Queen Mary University of London, London, United Kingdom; 4 Big Data Institute, Li Ka Shing Centre for Health Information and Discovery, Nuffield Department of Medicine, University of Oxford, Oxford, United Kingdom; 5 Department of Zoology, Peter Medawar Building, University of Oxford, Oxford, United Kingdom; 6 Rakai Health Sciences Program, Kalisizo, Uganda, School of Public Health, Makerere University, Kampala, Uganda; 7 School of Medicine, Makerere University, College of Health Sciences, Kampala, Uganda; 8 U.S. Military HIV Research Program, Walter Reed Army Institute of Research, Silver Spring, Maryland, United States of America; 9 Henry M. Jackson Foundation for the Advancement of Military Medicine, Bethesda, Maryland, United States of America; 10 Department of Medicine, Johns Hopkins School of Medicine, Johns Hopkins University, Baltimore, Maryland, United States of America; 11 Laboratory of Immunoregulation, Division of Intramural Research, NIAID, NIH, Baltimore Maryland, United States of America; Tel Aviv University, ISRAEL

## Abstract

In the absence of effective antiviral therapy, HIV-1 evolves in response to the within-host environment, of which the immune system is an important aspect. During the earliest stages of infection, this process of evolution is very rapid, driven by a small number of CTL escape mutations. As the infection progresses, immune escape variants evolve under reduced magnitudes of selection, while competition between an increasing number of polymorphic alleles (i.e., clonal interference) makes it difficult to quantify the magnitude of selection acting upon specific variant alleles. To tackle this complex problem, we developed a novel multi-locus inference method to evaluate the role of selection during the chronic stage of within-host infection. We applied this method to targeted sequence data from the p24 and gp41 regions of HIV-1 collected from 34 patients with long-term untreated HIV-1 infection. We identify a broad distribution of beneficial fitness effects during infection, with a small number of variants evolving under strong selection and very many variants evolving under weaker selection. The uniquely large number of infections analysed granted a previously unparalleled statistical power to identify loci at which selection could be inferred to act with statistical confidence. Our model makes no prior assumptions about the nature of alleles under selection, such that any synonymous or non-synonymous variant may be inferred to evolve under selection. However, the majority of variants inferred with confidence to be under selection were non-synonymous in nature, and in most cases were have previously been associated with either CTL escape in p24 or neutralising antibody escape in gp41. We also identified a putative new CTL escape site (residue 286 in *gag*), and a region of gp41 (including residues 644, 648, 655 in *env*) likely to be associated with immune escape. Sites inferred to be under selection in multiple hosts have high within-host and between-host diversity although not all sites with high between-host diversity were inferred to be under selection at the within-host level. Our identification of selection at sites associated with resistance to broadly neutralising antibodies (bNAbs) highlights the need to fully understand the role of selection in untreated individuals when designing bNAb based therapies.

## Author summary

During the within-host evolution of HIV-1, the diversity of the viral population increases, with many beneficial variants competing against each other. This competition, known as clonal interference, makes the identification of variants under positive selection a challenging task. We here apply a novel method for the inference of selection to targeted within-host sequence data describing changes in the p24 and gp41 genes during HIV-1 infection in 34 patients. Our method adopts a parsimonious approach, assigning selection to the smallest number of variants necessary to explain the evolution of the system. The large size of our dataset allows for the confident identification of variants under selection, alleles at certain loci being repeatedly inferred as under selection within multiple individuals. While early CTL escape mutations have been identified to evolve under strong positive selection, we identify a distribution of beneficial fitness effects in which a large number of mutations are under weak selection. Variants that were confidently identified under selection were primarily found to be associated with either CTL escape in p24 or neutralising antibody escape in gp41, including sites associated with escape from broadly neutralising antibodies. We also discovered four sites that are likely to be associated with immune escape. We find that the most frequently selected loci have high diversity both within-host and at the between-host level.

## Introduction

In the absence of effective antiretroviral therapy, HIV-1 evolves rapidly during infection. A key driver of evolution is the influence of the host immune system; cytotoxic CD8+ T-cells (CTLs) and neutralising antibodies (nAbs) impose selection on the virus, leading to the emergence of immune escape mutations[1]. However, other factors also influence viral evolution. For example, the host-specific nature of the immune response leads to the accumulation of mutations which are deleterious to the virus upon transmission to a new host. During the course of a new infection such variants are often lost, in particular where they occur at sites which in general are under strong purifying selection[2,3]. Selection may further act for protein or RNA secondary structure[4,5].

The complex nature of selection has led to multiple studies evaluating how the viral genotype may be both constrained and shaped during the course of evolution. These include the use of techniques for *in vitro* mutagenesis, and analyses of viral sequence data, evaluated at the level of population consensus or through deep sequencing exploring within-host variation at one or more time points during infection. For example, mutagenesis of HIV-1 proteins has allowed the measurement *in vitro* of the effect of specific mutations [6]. The development of technologies for high-throughput mutagenesis has enabled such measurements to be made across very large sets of potential mutations[7–9]. Mathematical methods combining such results have been used to generate an overview of fitness costs and epistatic effects for the virus[10]. Measurements of this form provide a base-level estimation of the general fitness landscape of the virus, although the extent to which *in vitro* data captures the behaviour of the virus in a human host may be limited.

Many years of study of HIV-1 have led to the collection of consensus genome sequence data for a large number of individual infections[11]. Such data have allowed techniques such as the fitting of maximum entropy models, which characterise the fitness costs of non-consensus variants in regions of the viral genome[12–14]. While the evolution of HIV-1 occurs in within-host environments that differ between individual hosts, these models provide something of a mean picture of the viral response across an averaged, within-host environment [15,16]. In these models the extent of conservation at a particular genetic locus indicates the extent to which purifying selection acts upon the majority allele[17].

Short-read deep-sequencing data has provided valuable insights into how fitness effects shape the evolution of HIV-1. Studies can be broadly categorised into those that consider purifying selection, and those that consider positive selection. Purifying (or negative) selection represents the process by which deleterious variants are purged from a population. Over time the frequency of a variant under purifying selection evolves in a statistically predictable way towards an equilibrium state via mutation-selection balance[18]. Exploiting this fact, allele frequencies observed over time during single untreated infections[2], or at single time points within multiple infections, have been used to estimate the magnitude of selection and the mutation rate acting upon distinct regions of the genome[17,19].

Positive selection represents the process by which favourable variants are driven towards fixation. As with purifying selection population genetic methods can be adopted for the inference of fitness effects. For example, a series of models have been developed for the inference of HIV-1 escape rates from CTL responses. Whereas earlier approaches to this problem considered viral escape from a single CTL response[19–22], more recent studies have considered the multiple immune responses that arise successively during infection [23–25]. Under such circumstances, interference between beneficial viral mutations affects the population dynamics [26]. Therefore, accounting for this clonal interference is critical if the role of selection is to be correctly inferred[27–29].

Studies assessing fitness effects in within-host HIV-1 infection have often focused upon the earliest stages of infection when strong selection on CTL escape mutations typically dominates the viral population dynamics[23,29]; in this circumstance, we can model evolution as a competition between a relatively small number of viral genotypes [23,30]. Later in infection, where escape mutations are less strongly beneficial, and where synonymous diversity has had longer to accumulate [2], the potential for hitchhiking and clonal interference is greater, such that variants observed at high frequency are less certain to have evolved under positive selection. In this circumstance, distinguishing selected from non-selected variants is a substantial challenge. To address this, we here present a *de novo* approach for inferring selection from HIV-1 sequence data in which any variant allele may, in theory, be detected as under selection. We adopt a parsimonious approach, assigning selection to the smallest set of variants required to explain the observed multi-locus sequence data under a likelihood model. Applied to targeted sequence data from a substantial cohort of 34 untreated individuals living in Uganda[31], we determine how selection drives viral evolution. In the presence of pervasive interference between alleles in linkage disequilibrium with one another, our consideration of data from a large number of individuals is fundamental in providing statistical confidence in the assignment of selection. Specifically, the repeated inference of selection at the same locus in different individuals enhances the power of our study to elucidate how selection during individual infections shapes genetic diversity at the population level.

## Results

We applied an evolutionary inference method to deep-sequencing data spanning multiple years of infection from 34 untreated individuals living in Rakai, Uganda, enabling us to infer positive selection acting on part of the gp41 region of *env* (324 base pairs) and the p24 region of *gag* (387 base pairs)[31].

### Extent of selection

**Fig 1:**
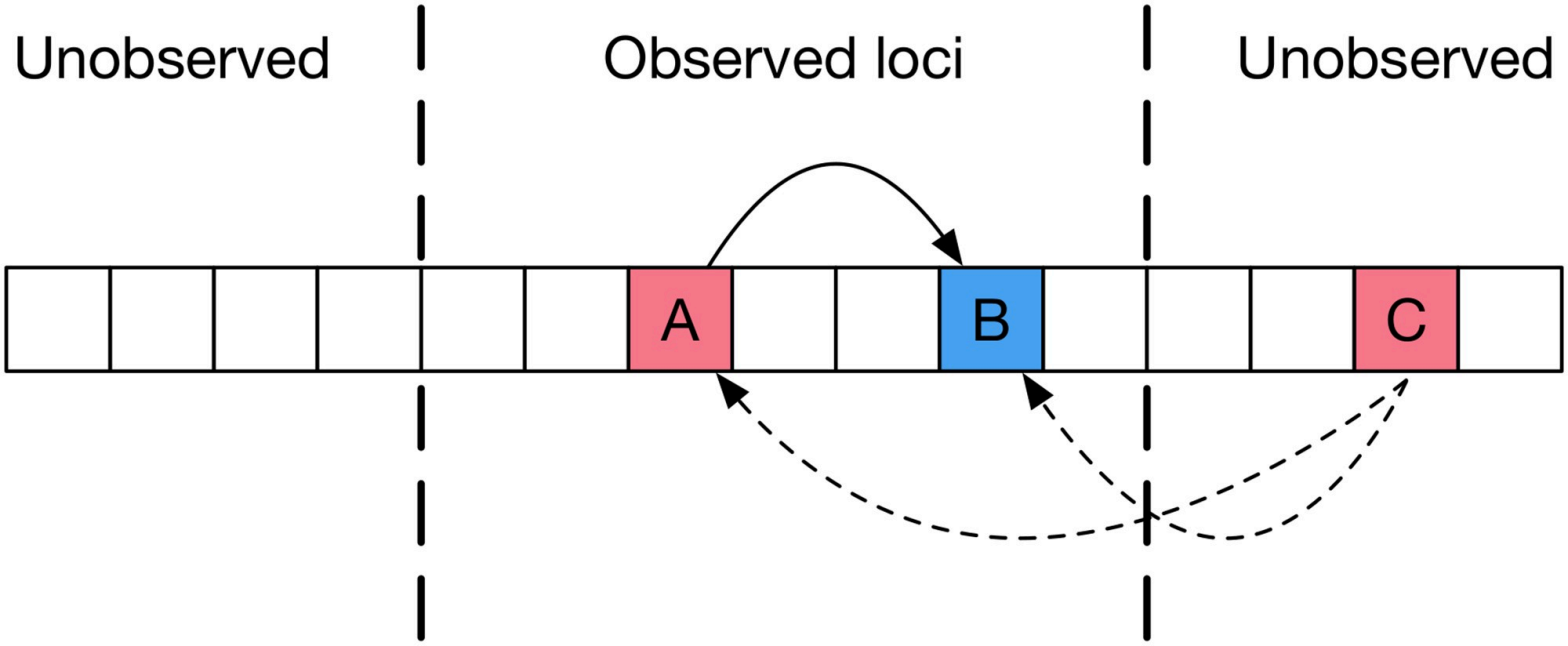
Linkage disequilibrium between alleles in the virus. Here positive selection at locus A affects the behaviour of alleles at locus B due to linkage. Our method of inference corrects for such effects if A is observed, whereas a single-locus method could lead to incorrectly inferring selection at B. However, in the system shown linkage disequilibrium also exists between alleles at A and B and those at the unobserved locus C. Such effects cannot be accounted for by our approach. We used a simulation-based approach to estimate the importance of such effects, so as to account for their influence upon the results generated by our method.

An initial application of our method found extensive evidence of positive selection in the viral genome, with 74 (out of 387) nucleotide sites in p24 and 81 (out of 324) nucleotide sites in gp41 being inferred to evolve under positive selection in at least one individual (S1 Table and S1 Fig). Our method explicitly accounts for linkage disequilibrium between alleles observed in the sequencing data[27,32]; a potential remains for interactions between observed alleles in the targeted sequence region, and non-observed alleles in flanking regions of the genome (Fig 1). To estimate the effect of these interactions on our results, we ran simulations to replicate the dynamics of real infections. In a multi-locus system where all alleles under selection are observed (i.e. in which the full data of the system are available), our approach performs very well, identifying the majority of variants under selection with very few false positive inferences. However, when non-observed selected alleles interact with observed alleles via linkage disequilibrium (i.e. where only partial data of the system is available), our approach is prone to generating false positive inferences of selection (S1 Text, S3 and S4 Tables). Data from our simulation study allowed us to set statistical criteria via which we could combine inferences from multiple patients and confidently identify sites under selection, despite the presence of false positive calls.

### Strength and time of onset of selection

**Fig 2:**
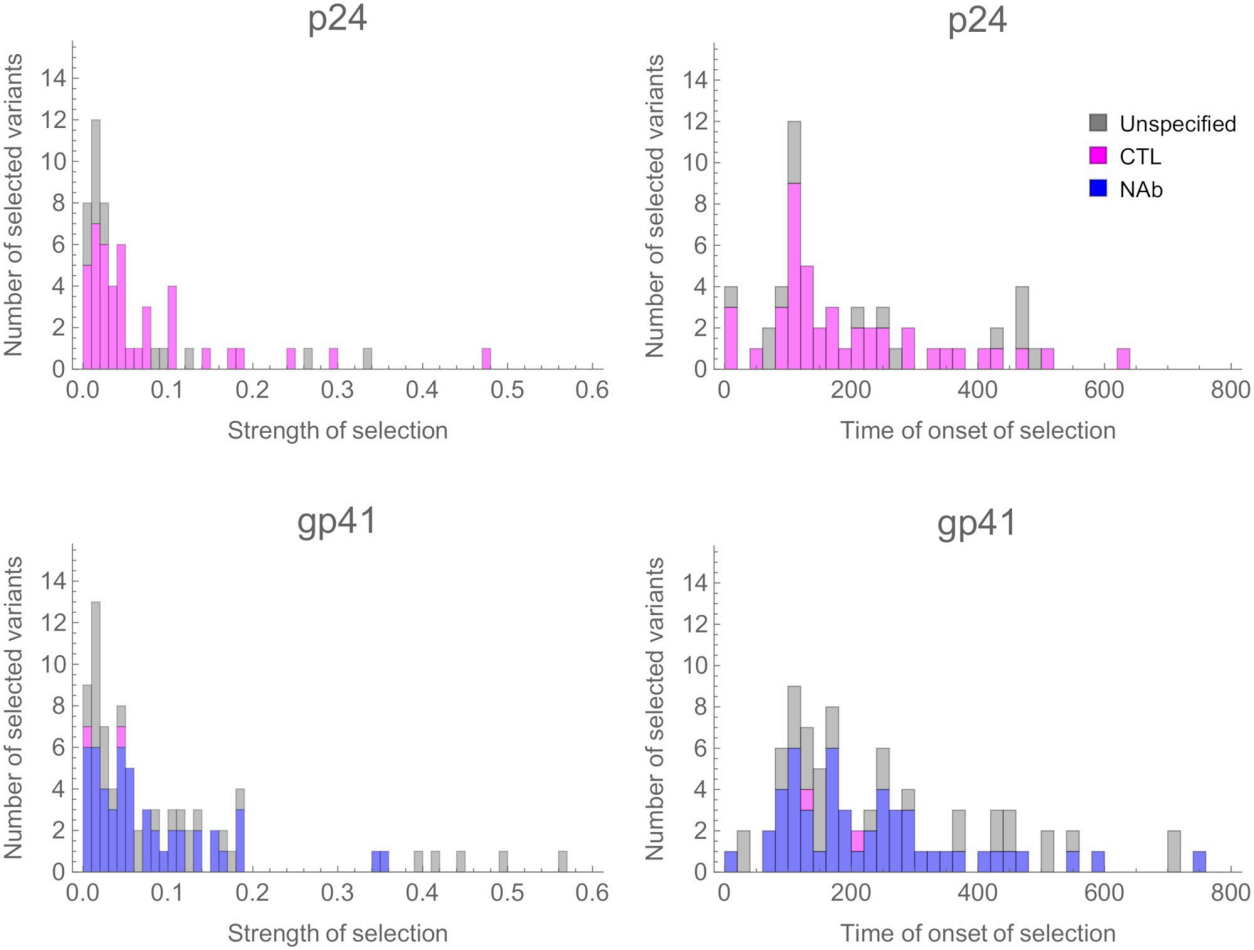
Histograms of inferred strengths of selection and times in days of onset of selection across 34 individuals. The maximum likelihood estimates are shown in each case, for the subset of the data where the upper and lower bound confidence intervals on the strength of selection are within an order of magnitude of each other. Bars indicate variants at nucleotide sites, and are coloured according to whether they are within AA positions associated with differences in susceptibility to CTLs (pink) or susceptibility to NAbs (blue). Where a codon is implicated in both a NAb and CTL response, for clarity it is coloured blue (see S2 Table).

Inferred variants generally evolved under weak selection. In constructing a distribution of the strength of selection among selected variants, we first assessed the degree of precision with which this statistic could be inferred at each locus. The uncertainty in a given estimate depends upon the extent to which data are available. For example, when a variant emerges and fixes between two time points, only a lower bound on the strength of selection can be inferred[33]. We therefore generated confidence intervals for each inferred magnitude of selection using a likelihood-based method, retaining only variants for which the upper and lower bounds of this interval differed by no more than an order of magnitude. Distributions of fitness effects compiled from these variants showed that most of the identified alleles under selection experienced very weak selective effects, with long tails of alleles evolving under strong positive selection (Fig 2). Application of our method to simulated data highlighted an undercalling of very weakly selected variants, and an underestimation of the magnitude of selection affecting the strongest variants (S2 Fig), however the overall inferred distribution of selection coefficients was not statistically different to the ‘true’ distribution used to generate simulations (S3 Fig). Further statistical details and notes on these simulations are provided in S1 Text, S4, S5 and S6 Figs.

In p24, around 65% of inferred selected variants had a strength of selection of less than 5% per generation, while in gp41 approximately half of variants were under this threshold. In our model, selection of 5% per generation would cause, in the absence of interference effects, a change in allele frequency from 5% to 95% in a period of just under eight months. Most selection acting upon variants was inferred to kick in during the first year of infection and almost all within two years, and we found no evidence of a correlation between the strength and time of onset of selection (S7 Fig). Our approach underestimates the proportion of variants under weak selection; very weak selection would not produce a change in the population sufficient to be identified from the data. The extreme beneficial end of the distribution may also be under-represented because variants which fixed within individuals before the first sample was collected cannot be identified from our data. Finally, the finite period of time over which sequencing was performed could restrict the inference of more lately selected variation because a selected variant arising later in infection would have less time to affect the composition of the population in an observable way.

### Distribution of selected variants among individuals

Using the simulated data, we identified a statistical threshold at which we could robustly identify specific sites in the genome containing variants under selection despite the presence of false positive inferences of selection. A unique aspect of this dataset is the large number of untreated individuals included in the study. Where alleles at the same locus were inferred to be under selection in multiple individuals, a statistical approach was used to infer loci at which, under conservative assumptions, at least one of the inferred variants is genuinely under selection. Taking into account different patterns for nonsynonymous and synonymous mutations, we estimated per-site false-positive rates for nonsynonymous and synonymous mutations, which were subsequently used to identify with statistical confidence sites that were under selection in at least one individual in our dataset. The process used for estimation is described in full in S1 Text. Taking all 34 individuals into consideration, we calculated that in p24 we could be confident that a site is under selection in at least one individual if mutations were inferred to be under selection in at least five individuals, and/or if nonsynonymous mutations were inferred to be under selection in two or more individuals, while in gp41 we could be confident if at least five mutations and/or three nonsynonymous mutations were inferred to be under selection. This is a conservative approach, and will exclude sites genuinely under selection in only one or a few individuals, for example sites associated with escape from rare HLA alleles or NAbs, or other less common forms of selection.

**Fig 3:**
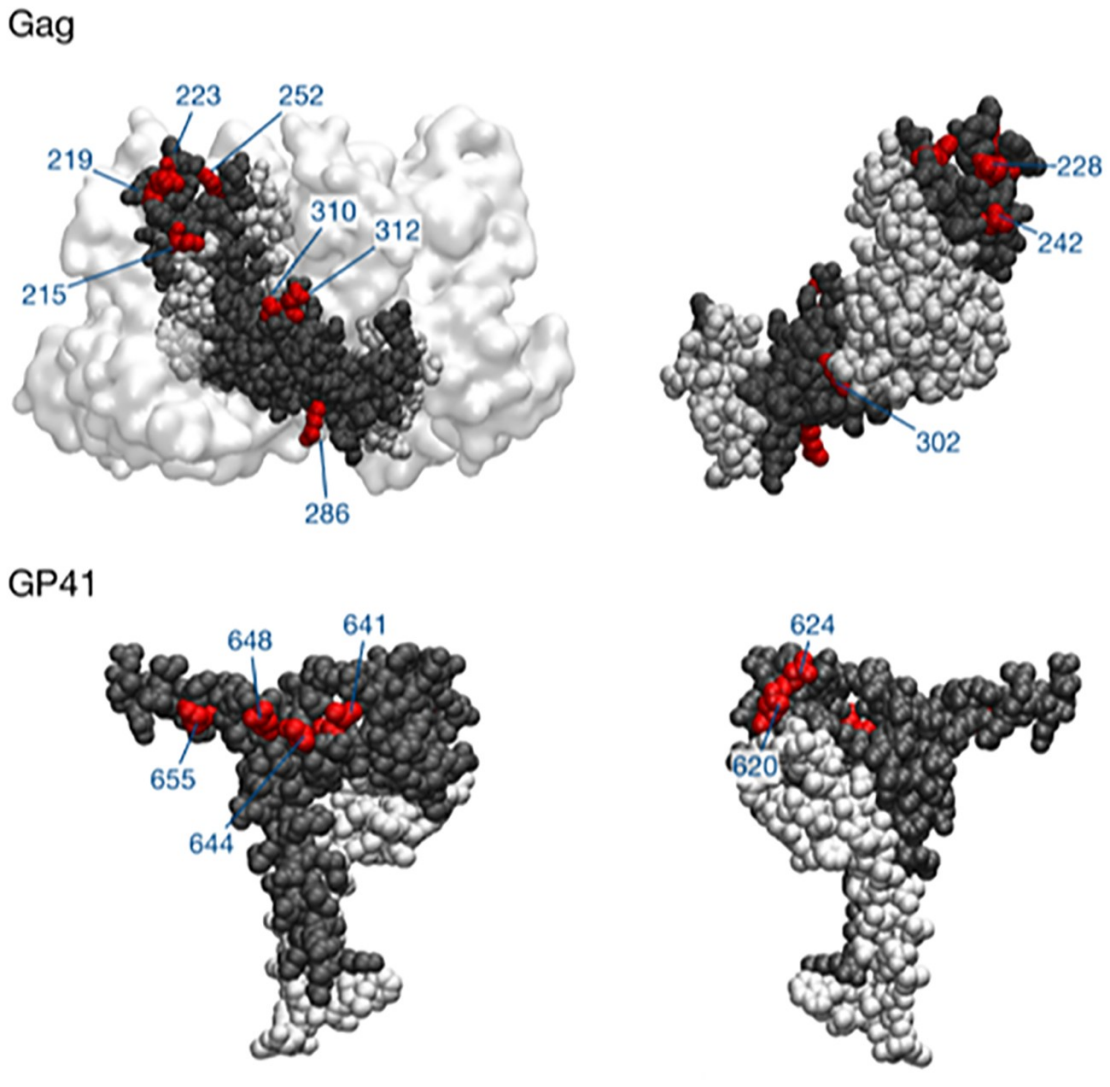
Locations of selected positions on the protein structures of p24 and gp41. Protein structures with pdb identities and 3J34 and 6NIJ were used to plot these figures [103,104]. Parts of proteins that were covered by sequence data are shown in gray, with the remainder of the protein in white van der Waals representation. Amino acid positions identified by our analysis are shown in red. The gp41 structure does not show the entirety of the sequenced region, with amino acids at positions 670 and 674 also identified in our analysis, but not in the protein structure. Other nearby proteins are shown in white surface representation. Figures were created using the VMD software package [105].

Applying these criteria, we identified 11 specific nucleotide sites, representing 10 amino acid (AA) positions, under selection in p24, and likewise we identified 11 such sites, representing 8 AA positions, in gp41. All but four mutations at these AA positions represented nonsynonymous changes (see Table 1). Occasionally two different codons at the same AA position were found to be subject to selection in a single individual, but in general repeated inferences of selection at an AA position occurred in distinct individuals (S1 Table). The positions of the identified mutations in their respective protein structure are shown in Fig 3.

**Table 1:** Summary of amino acid positions containing sites under selection.

Region	Amino Acid Position^a^	Sensitivity^b^	Nonsynonymous^c^			Synonymous
			Reversion	Escape	Neither	
p24	215[34–37]	CTL	0	2	1	2
p24	219[37–40]	CTL	1	0	1	0
p24	223[37–40]	CTL	2	4	2	0
p24	228[39,40]	CTL	1	1	0	0
p24	242[40–45]	CTL	2	0	1	0
p24	252[36,40]	CTL	1	2	0	0
p24	286		1	1	0	0
p24	302[46]	CTL	2	0	0	0
p24	310[47–50]	CTL	1	1	0	0
p24	312[36,46,48,51,52]	CTL	3	2	0	0
gp41	620[53–57]	NAb	3	3	4	1
gp41	624[53,54]	NAb	3	2	4	0
gp41	641[58]		0	2	1	1
gp41	644[58]		2	0	1	0
gp41	648[58]		1	3	0	0
gp41	655[59]	NAb	1	3	0	0
gp41	674[56,60–65]	NAb	0	2	1	0
gp41	677[56,62,63]	NAb	1	3	0	0

^a^Amino Acid (AA) positions in *gag* (p24) or *env* (gp41) relative to the HXB2 reference genome

^b^Sensitivity to CTLs or NAbs using the Los Alamos HIV database (http://www.hiv.lanl.gov; see Methods for further details). AA position 228 in *gag* is associated with compensatory mutations, rather than affecting sensitivity to CTLs directly. Position 286 in *gag* occurs in an epitope position targeted by common HLA alleles in the Ugandan population and so is likely associated with CTL escape. Positions 644, 648 and 655 were not associated with sensitivity to NAbs, but are in an epitope region recognised by NAb HGF24 in some viruses isolated from Africa[58].

^c^A nonsynonymous change was classed as a reversion if the nucleotide changed towards the subtype-specific population-level consensus, as an escape if the nucleotide changed away from the subtype-specific population-level consensus, and as neither of these if there was a nucleotide change which remained different to the subtype-specific population-level consensus.

**Fig 4:**
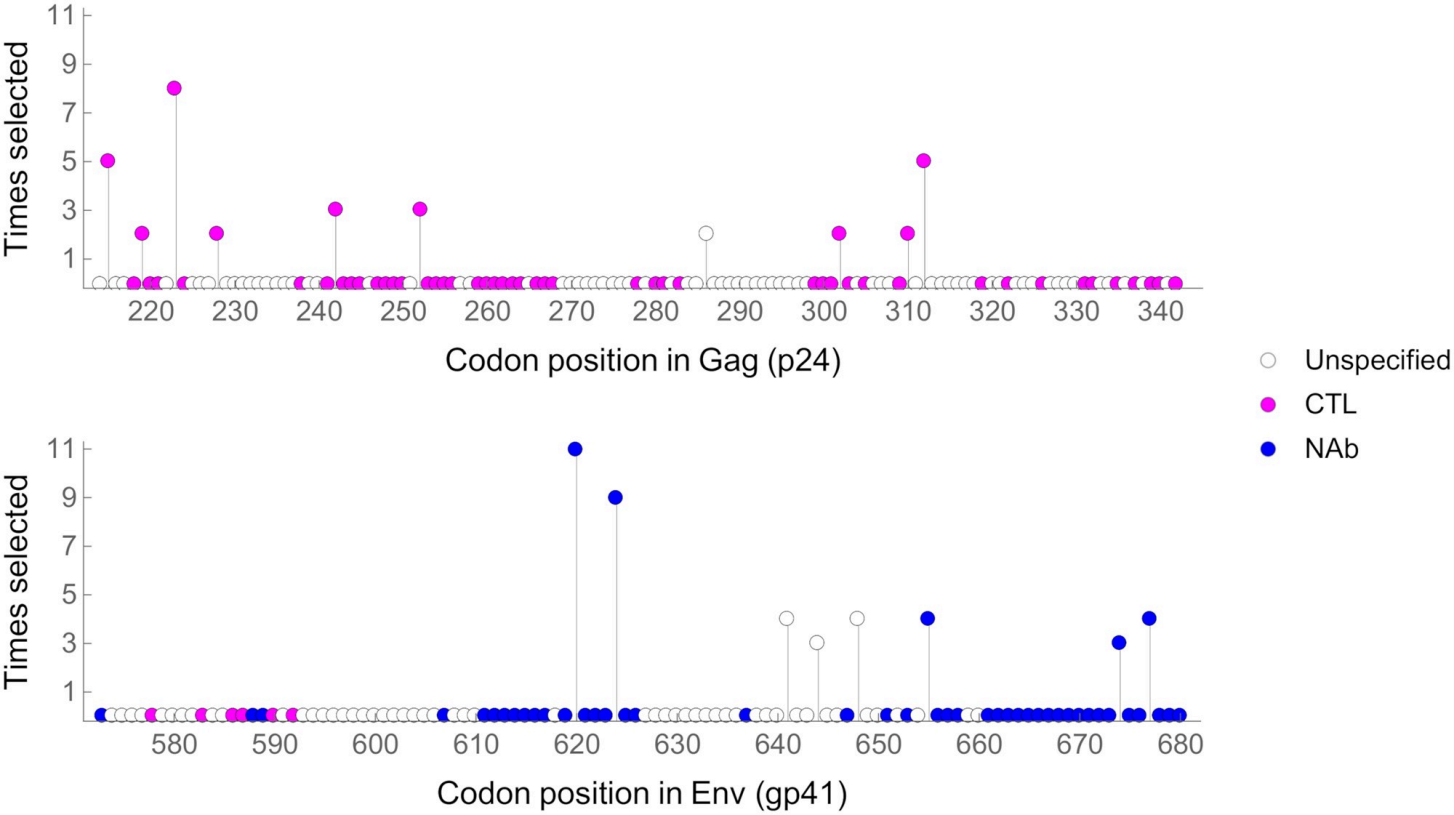
Amino Acid (AA) positions containing nucleotide sites that with confidence were inferred to be under selection in at least one individual. Codon positions are in relation to the HXB2 reference sequence and the vertical y-axis gives the number of times that codon was inferred to be under selection across 34 individuals. Occasionally the same AA position was inferred to be under selection twice in the same individual. Pink: AA positions associated with changes in susceptibility to CTLs or compensatory mutations; Blue: AA positions associated with susceptibility to NAbs. Selection at Gag residue 286 possibly reflects CTL escape in one individual and reversion in another. Selection at Env residues 641, 644, and 648 possibly reflect selection associated with NAbs; these are all within an epitope region targeted by the neutralising antibody HGF24 in some African Isolates[58]. Where an AA position is implicated in CTL and NAb responses, for clarity they are coloured in blue.

Using the Los Alamos HIV database (http://www.hiv.lanl.gov), for all of the AA positions in our study, we determined whether they have previously been associated with changes in CTL susceptibility or NAbs (see Methods, Fig 4, S2 Table). In p24, 43% of sites have previously been associated with changes in CTL susceptibility and/or compensatory mutations, whilst 69% of the variants we inferred to be under selection were associated with CTL and/or compensatory mutations. For the 10 AA positions identified as almost certainly under selection, nine have previously been associated with changes in CTL susceptibility and/or compensatory mutations, and lie in epitope regions recognised by multiple HLA alleles present in the Ugandan population[66–68]. The remaining codon (residue 286 in HXB2 *gag*) lies within CTL epitopes recognised by human leukocyte antigen (HLA) alleles that are relatively common in the Ugandan population (A1101 and B27[66]) and therefore mutations at this site probably affect sensitivity to CTLs, or possibly compensatory mutations. In gp41, 44% of AA sites have previously been associated with NAbs, whilst 61% of the variants we inferred to be under selection were associated with NAbs. Of the eight AA positions identified as being almost certainly under selection, five (residues 620, 624, 655, 674 and 677 of *env*) have previously been associated with NAbs, including broadly neutralising antibodies (bNAbs). These are associated with the gp120/g41 interface and the membrane-proximal external region (MPER). The other three codons (residues 641, 644 and 648 of *env*) all lie within an epitope region that is targeted by the monoclonal antibody HGF24, which has been shown to neutralise some viruses isolated from the African continent[58]. Although we cannot rule out other sources of selection in *env*, given the position of these codons on the genome, we believe selection due to humoral immune pressure is more likely. As such, all of the sites we identified as under selection in p24 probably affect susceptibility to CTLs or associated compensatory mutations, whereas all of the sites under selection in gp41 probably affect susceptibility to NAbs or associated compensatory mutations.

### Direction of selection

Since mutations that enable the virus to evade host immune responses are often costly in terms of viral replication[69,70], sites harbouring escape mutations are likely to evolve towards the population-level consensus when transmitted to a new host since the selective pressure of a specific immune response is removed, but costs associated with the mutation remain [2,31,71–74]. This hypothesis is supported by the observation that allelic substitutions during the course of untreated infection occur towards population consensus much more frequently than expected by chance [2,31]. If analysis is restricted to sites where substitutions are observed, this bias is confined mainly to nonsynonymous substitutions[31], which is expected if immune escape mutations are generally nonsynonymous.

Since the HLA types of the 34 individuals in our study are unknown, we followed previous studies by classifying variants we confidently inferred to be under selection as escapes or reversions depending on the population-level consensus[2,71]. Specifically, we classified selected variants as escapes if they resulted in an AA change away from the subtype-specific population-level consensus in Uganda during the period the individuals were being sampled (see Methods for full details). Conversely, inferred selected variants were classified as reversions if they resulted in change towards the subtype-specific population-level consensus. Of the 32 selected nonsynonymous mutations identified in p24, 13 were classified as escapes, 14 as reversions, and five as neither. The similar number of escapes and reversions observed is expected if CTL immune escape mutations are costly in non-HLA matched hosts, with escape in one individual followed by reversion in the next. The observation of one escape and one reversion in residue 286 of *gag* supports our prediction that this locus likely affects CTL susceptibility. Of the 40 nonsynonymous mutations identified in gp41, 18 were classified as escapes, 11 as reversions, and 11 as neither. The fact that over a quarter of AA changes are towards population consensus suggests that in many cases antibody escape mutations are deleterious in hosts without a matching antibody, but the stereotypical pattern of adaptation in one individual followed by reversion in another found at CTL immune epitopes is likely more complex for antibody-escape evolution, where escape mutations are sometimes, but not always costly in the absence of an antibody response [75,76]. Among the three residues 641, 644 and 648 of *env*, five of the nine nonsynonymous variants are escapes, three are reversions, and two are neither, supporting our prediction that these sites affect susceptibility to host (probably NAb) immune responses.

We also determined the direction of change for all variants inferred to be under selection, which will likely include variants under direct selection and variants changing in frequency due to hitch-hiking, and whether they represented synonymous or nonsynonymous changes (S8 Fig). We found a clear correlation between the number of times that selection was inferred at an AA position and the proportion of selected variants that were nonsynonymous (linear regression, p24 p = 0.009, r^2^ = 0.85; gp41 p = 0.007, r^2^ = 0.67). For gp41, a correlation was also found between repeated selection at an AA position and a pattern of evolution towards the population consensus (p = 0.011, r^2^ = 0.63); around half of inferred selection events were towards population consensus at the AA position most frequently inferred to be under selection. Although for p24 the linear regression did not reveal a significant trend (p = 0.400, r^2^ = 0.18), there is a distinction between AA positions selected 2 or more times, which have a high probability of being towards subtype-specific population consensus (44%), and AA positions selected once, which have only a small probability of being towards the consensus (5%). Our interpretation is that AA positions represented once in our analysis disproportionately represent mutations increasing in frequency due to hitch-hiking, with these mutations tending to be synonymous and away from population level consensus. AA positions represented multiple times, on the other hand, are more likely to represent immune escapes and reversions, and therefore tend to be nonsynonymous but with only around half of mutations away from consensus.

### Comparing codon diversity at the within-host and population scale

**Fig 5:**
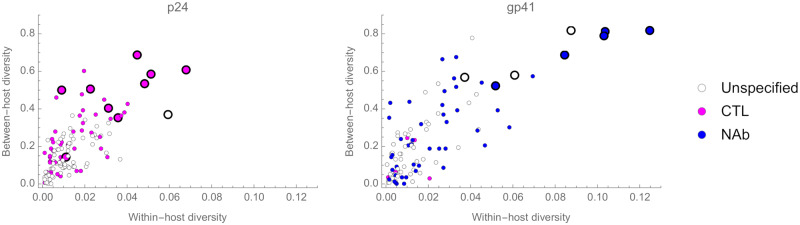
Within- and between-host codon diversity. For every AA position in our region of analysis we determined the within- and between-host codon diversity. Large markers denote AA positions in which we are confident selection is occurring in at least one individual. Markers are coloured if they are associated with changes in sensitivity to CTLs or compensatory mutations (pink) or NAbs (blue). The site confidently under selection in p24 but which is unspecified (Gag residue 286) is possibly associated with CTL escape. The sites confidently under selection in gp41 but which are unspecified (Env residues 641, 644, and 648) possibly reflect selection associated with NAbs; these are all within an epitope region targeted by the neutralising antibody HGF24 in some African Isolates[58]. Where an AA position is implicated in CTL and NAb responses, for clarity they are coloured in blue.

Our data showed a strong relationship between within- and between-host sequence diversities, where the diversity of codons was measured at each AA position. Within-host diversity was measured approximately three years after seroconversion for each of the 34 individuals, and the mean calculated. Diversity at the population scale was calculated as the mean of the diversities for each of the subtypes A, D, and C, using virus sequences from a large number of individuals living in Uganda around the same time as the 34 individuals in our study (see Methods). Consistent with previous studies [2], we identified a strong relationship between measurements of sequence diversity calculated at the within-host and population scales (Fig 5, S2 Table), with all AA positions found to be highly diverse at the within-host level also highly diverse at the population level. Moreover, all but one of the AA positions containing nucleotide sites that we are confident are under selection are also diverse at the population level (the exception being residue 302 in *gag*). Given that most changes at these sites probably reflect escape from host immune responses, compensatory mutations, or reversions of these escapes in subsequent individuals, diversity at the population scale at these AA positions is likely maintained by the differing selection pressures faced by variants in different hosts due to different immunological backgrounds.

Not all AA positions found to be highly diverse at the population level were detected as being under selection, with a high degree of confidence, at the within-host scale. This observation could arise if codons at these positions are under selection in some individuals in the population, but we failed to observe it with confidence, either because we sampled too few individuals, selection was too weak to be detected, and/or selection drove fixation events before the first sampling time point. In addition, at unconstrained sites experiencing little or no selection, diversity might gradually accumulate at the population scale due to drift, exacerbated by small bottleneck sizes at transmission. This could explain the high levels of population diversity at positions 235 in *gag* and 609 in *env*; almost all of the diversity observed at these codon positions is due to the presence of synonymous variants.

## Discussion

We developed a novel inference framework to infer the extent of selection acting upon variants which drive the evolution of within-host HIV-1 populations, considering data from the p24 region of *gag*, and gp41 of *env*, from 34 longitudinally sampled untreated individuals. A frequent assumption is that beneficial mutations will rapidly spread within individuals once they occur[77]. For example, it is well established that CTL-escape mutations accumulate and spread rapidly during acute infection [19–25], though that the rate of allele fixation decreases during chronic infection[22,25]. However, estimating the extent and strength of positive selection during infection more generally is challenging due to genetic linkage among variant alleles, which makes differentiating between selected variants and variants that are increasing in frequency due to linkage with a selected variant (hitch-hiking) difficult. Our *de novo* approach incorporates genetic linkage and recombination. Furthermore, it is generally assumed that during untreated infection selected variants are associated with immune escape; our approach is agnostic with regards to phenotypic data, potentially allowing any polymorphic site in the genome to be identified as under positive selection. Indeed, using our approach we discovered four sites in the genome likely to be under immune pressure (both CTL and antibody) that were not listed in the Los Alamos database.

Our results indicate a pattern of weak and slow selective sweeps characterising evolution during chronic HIV-1 infection, with stronger faster selective sweeps being relatively rare. We note that where ‘weak’ selection was inferred, this was still on a scale outweighing the effects of genetic drift. Studies of the effective population size of HIV-1 have indicated a value in excess of 10^5^ [76]; given such a value, selection of the order of 5% per generation is comfortably within a realm whereby the influence of selection dominates that of genetic drift [78]. An important caveat is that the first sampling time point for each individual in our analysis is estimated to be between 150 and 425 days since seroconversion, and therefore we will not detect variants that were under strong selection and rapidly reached fixation before the first sampling time point. Furthermore, the magnitude of the most strongly selected variants could not always be quantified; where fixation occurs entirely in the interval between two consecutive time points, no upper bound on the magnitude of selection could be fixed.

A unique aspect of our study is the large number of individuals for which we have data. Comparisons among individuals revealed AA positions which were inferred to be under selection in multiple (up to ten) individuals. Most of these sites have previously been identified as affecting sensitivity to CTLs (in p24) or NAbs (in gp41), with selected changes at these sites likely reflecting the gain or loss immune-escape mutations, or escape-related compensatory mutations (although other sources of selection cannot be ruled out). In addition, we identified four AA positions under selection in multiple individuals that haven’t previously been identified as affecting sensitivity to CTLs or NAbs (residue 286 in *gag*, and residues 641, 644 and 648 in *env*). Given the patterns of selection at these sites, reflecting evolution both away and towards population consensus, it is likely these sites also affect sensitivity to CTLs (*gag*) or NAbs (*env*). Sites under selection in multiple individuals were also found to be highly diverse at the population level. This again is consistent with a pattern where a minority of codons are repeatedly under selection, likely representing adaptation to the immunological background of some individuals, which revert upon transmission to subsequent individuals; a pattern which has been referred to as “adapt and revert”[79]. Although we do not know the HLA-type of the infected individuals in our study, the number of putative CTL escapes and reversions is consistent with the frequency of different HLA alleles in the Ugandan population.

Perhaps less expected in our analysis was the identification of AA positions that are associated with NAbs and which were found to be under selection in a large number of individuals; for one site selection was inferred in nearly a third of individuals, with another inferred in a quarter of individuals. The implication is that the same epitopes are frequently targeted by antibodies in different individuals, and with similar means of viral escape. Moreover, since around a quarter of changes at these sites are towards the subtype-specific population level consensus, many may well represent the reversion of costly antibody-escape mutations from previous individuals, supporting the observation that some but not all antibody-escape mutations are costly [70,75,80–84]. These patterns can help explain why resistance to antibodies has increased over the course of the epidemic [85–89], but also highlights that viral evolution at the population level in response to bNAb-based interventions is likely to be complex, involving evolutionary responses to both naturally and therapeutically induced immune responses.

Even though our framework explicitly accounts for linkage disequilibrium between observed variants, it is still vulnerable to false positive inferences of selection due to linkage disequilibrium with unobserved variants flanking the genetic regions we analysed. Although simulated data suggested that the overall distribution of fitness effects was robust to this vulnerability, our study should serve as a cautionary note; where multiple alleles evolve in linkage disequilibrium, care is needed in identifying selection with any particular allele. The large number of individuals included in our study enabled us to partly circumvent this problem by only assigning confidence that any particular nucleotide site is under selection if it is inferred to be under selection in multiple individuals. Indeed, evidence for the validity of our method is provided by the repeated observation of variants under weak selection across multiple individual infections, with these changes making biological sense under the “adapt and revert” hypothesis. Our results emphasise the role of immune escape in driving evolution during chronic infection, shaping patterns of diversity at the population level, and provides new insights that could be useful in the development of immune-based interventions, particularly in the context of viruses circulating in Africa.

## Methods

In order to evaluate selection within a host, we employed a likelihood-based inference framework to infer the most parsimonious explanation of the sequence data in terms of a model of selection acting for specific nucleotides in the viral population. Some of the mathematical aspects of this framework have previously been applied in studies of the within-host evolution of the influenza virus[32,90], although the details of the model used here tailor it to HIV-1 infection. Our model explicitly accounts for linkage disequilibrium between alleles and builds upon earlier approaches for inferring selection in cases where linkage is of importance for evolution [23,27,91,92].

### Model outline

Our model proceeds through a number of steps (see below for full details). (1) We identified variant alleles from the sequence data using a simple frequency cutoff. Measurements of the frequencies of variant alleles over time were collected into trajectories, each trajectory describing the frequency of a single allele over time. (2) Sets of alleles with similar trajectories were identified, and under the assumption that all of the differences between these similar trajectories resulted from noise in the data, the extent of noise, modelled as a single parameter, was estimated. The noise parameter defines a likelihood function for the data, the existence of which allows for fits to be made between the data and a number of models describing the evolution of the population. (3) Models of evolution at a single locus were used to assess all of the derived trajectories. Comparing the likelihoods of models describing evolution under selective neutrality, and under selection, we identified, using model selection, a subset of trajectories which potentially evolved under non-neutral selection. Such selection could arise either from intrinsic selection for or against the allele, or via linkage disequilibrium with an intrinsically selected allele elsewhere in the genome. (4) We combined the alleles present at each locus that had a potentially non-neutral trajectory into haplotypes, with these haplotypes only describing alleles at the loci identified to have potentially non-neutral trajectories (S9 Fig). (5) For each patient, the number of reads with each observed haplotype at each time of sampling was counted, forming a multi-locus dataset describing the evolution of the virus in that patient over time. (6) A series of multi-locus evolutionary models were fitted to the data from each patient, identifying the most parsimonious explanation of the data in terms of time-dependent selection acting on individual alleles in each dataset. (7) Confidence intervals for the inferred selection parameters were generated, again making use of the likelihood function. The model is thus identical to that described in a previous publication [33] with differences only in the approach to identifying noise in step two, in the models used to fit the data in step six, and in the calculation of confidence intervals for selection.

### Sequencing data

For our evolutionary analysis we used previously generated deep-sequence data from 34 longitudinally sampled individuals participating in the Rakai Community Cohort study and co-enrolled in the Molecular Epidemiology Research (MER) seroconverter study. Targeted short-read deep-sequence data from the p24 region of gag (390 bp; HXB2 reference genome positions 1429–1816) and the gp41 region of env (324 bp; HXB2 7941–8264) had been sequenced using the 454 sequencing platform (Roche, Branford, CT). All individuals were untreated, with a first sampling time point around one year since seroconversion, and typically 3 or 4 subsequent time points spanning between two and seven years of infection (see Table 1 in Raghwani et al 2019). Aligned sequences can be found at https://github.com/katrinalythgoe/

RakaiHIV. Further details on the individuals, including viral loads and CD4 counts, and sequencing methods used have been given elsewhere[31,93].

### Calling of variant alleles and trajectories from sequence data

Single-locus variants were identified in the data using the SAMFIRE software package[94]. Variants with a minimum allele frequency of at least 1% for at least one time point in the course of infection were identified. Variant frequencies collected over time were described in terms of trajectories. By way of notation, in a given patient we denote the trajectory

$$(i,a)={\left\{{\tilde{q}}_{i}^{a}(t_{k})\right\}}_{k=1,\ldots ,K}$$

comprising the observed frequencies of allele *a* at locus *i* across all recorded times *t*_*k*_. Here *K* is the total number of points in time at which the population was observed via sequencing, which varied between 2 and 5. We note that the frequency is calculated simply as, the number of observations of allele *a* at locus *i* at time *t*_*k*_ divided by the total number of alleles observed at locus *i* at time *t*_*k*_.

### Estimating the extent of noise in the data

Noise in sequence data can arise either through the collection of an unrepresentative sample of viruses from a patient, or via errors induced in the experimental processing and sequencing of that sample [93]. We here applied a heuristic method to derive a conservative estimate of the extent of noise in the data from the data itself. This was achieved by exploiting effects caused by genetic hitch-hiking[95]. If two alleles appear uniquely upon a shared genetic background, they will initially share an identical allele frequency. Over time the allele frequencies will change in a very similar manner, differences arising over time as a result of recombination between distinct haplotypes. We thus identified putatively hitch-hiking trajectories to derive an estimate of noise in the sequence dataset.

For this analysis, we considered loci at which a minor allele frequency of at least 10% was observed in samples collected at two points in time. Loci in HIV can potentially have multiple alleles satisfying this condition. For each pair of such loci, *i*, and *j*, we found the alleles a*, b* minimising the statistic

$$d_{ij}^{a^{*}b^{*}}=\underset{a,b}{\text{min}}\left\{\frac{1}{K}\sum_{k}\left|{\tilde{q}}_{i}^{a}(t_{k})-{\tilde{q}}_{j}^{b}(t_{k})\right|\right\}$$

where the minimisation was calculated over all polymorphic alleles at the loci *i* and *j*. Initially, pairs of trajectories (i,a*) and (j,b*) were denoted as being ‘similar’ if

$$d_{ij}^{a^{*}b^{*}}<10\%.$$

A set of further heuristic steps was then applied to refine these sets of trajectories. On timescales close to those over which the data for this study was measured, recombination in HIV-1 has been noted as being of importance over genetic distances greater than 100 nucleotides [2]; here a distance cutoff of less than or equal to 50 nucleotides was imposed between trajectories, that is |i-j| ≤ 50.

Next, under the assumption that pairs of our variants initially arose on the same background, pairs were required to have similar frequencies at the first time of observation, requiring that

$$|{\tilde{q}}_{i}^{a^{*}}-{\tilde{q}}_{j}^{b^{*}}|<5\%.$$

Further, so as to remove pairs of trajectories for which only one was polymorphic at a given time, it was required that the maximum ratio between minor allele frequencies did not exceed 10^3^ for the duration of the trajectories; the framework of a Dirichlet multinomial model we subsequently use to estimate the extent of noise does not perform well on very low frequencies. Remaining sets of pairs identified in each patient were clustered into sets via an iterative process; beginning with an initial pair of trajectories *(i*_*1*_, *a*_*1*_*)* and *(i*_*2*_, *a*_*2*_*)*, a trajectory *(i*_*m*_, *a*_*m*_*)* was added to the set if, following filtering, *(i*_*m*_, *a*_*m*_*)* was similar to a trajectory *(i*_*n*_, *a*_*n*_*)* already in the set. Sets of trajectories generated by the above process are shown in S10 Fig.

Having identified sets of trajectories, an inference process was used to evaluate the extent of noise in the data. Conservatively, the ‘true’ allele frequencies of each set were calculated as a simple mean of the observations, thereby assuming that all differences in frequencies result from ‘noise’ in the sequencing process. Given a set of trajectories *S* from a single viral population, recalling that ${\tilde{q}}_{i}^{a^{*}}(t_{k})$ was calculated as $n_{i}^{a}(t_{k})/N_{i}(t_{k})$, the inferred frequency at time *t*_*k*_ was calculated as the mean fraction of variant alleles across all loci with trajectories in *S*. That is,

$$q^{S}(t_{k})=\frac{\sum_{m}n_{i_{m}}^{a_{m}}(t_{k})}{\sum_{m}N_{i_{m}}(t_{k})}$$

A Dirichlet multinomial model was then parameterised across all trajectory sets, finding the value of *C* satisfying

$${\text{min}}_{C}\left\{\sum_{s}\sum_{m}\sum_{k}L(N_{im}(t_{k}),C,q^{S}(t_{k}),n_{i_{m}}^{a_{m}})\right\}$$

Where the summations are, respectively, calculated over sets of similar trajectories *S*, trajectories *m* in each set *S*, and samples *k* in each trajectory, and where the likelihood is the Dirichlet multinomial function

$$L(N,C,q,n)=\text{log}\frac{\Gamma (N+1)}{\prod_{a}\Gamma (n^{a}+1)}\frac{\Gamma (\sum_{a}Cq^{a})}{\Gamma (\sum_{a}n^{a}+Cq^{a})}\prod_{a}\frac{\Gamma (n^{a}+Cq^{a})}{\Gamma (Cq^{a})}$$

in which the sums and products with index *a* are calculated over all alleles at a specific locus, or equivalently over all haplotypes within a population, while G indicates the gamma function.

$$\Gamma (z)=\int_{0}^{\infty }x^{z-1}e^{-x}dx$$

The derived value of *C* provides a proxy measurement of the extent of noise in the data and was used in further likelihood calculations; we inferred the value *C* = 100.359. Qualitatively, this value represents the extent to which the variance of a sample of haplotypes is overdispersed in relation to a simple multinomial sample; a small value of *C*represents an increased amount of uncertainty in the data, tending towards a uniform distribution, in which samples are fully uninformative, while a high value of *C* represents increased uncertainty, tending towards a multinomial sample in which every read precisely identifies a haplotype in a perfectly representative sample from the population. We note that patterns of noise in genome sequence data may be substantially more complex than represented by our model; our likelihood, combined with the BIC model selection framework, provides a simple yet analytically tractable approach for the inference of selection parameters from real genome sequence data.

### Identification of potentially non-neutral loci

Considering frequency data from each variant allele, ‘potentially non-neutral’ loci [92] at which significant changes in allele frequency were observed over time were identified. In this process, where *q*^*1*^*(t)* denotes the frequency of the variant allele at locus *i* at time *t*, deterministic models of evolution at a single locus were fitted to the single-locus trajectories, using the equation

$$q_{i}^{1}(t_{k+1})=\frac{q_{i}^{1}(t_{k})e^{\sigma_{k}(t_{k+1}-t_{k})}}{1-q_{i}^{1}(t_{k})+q_{i}^{1}(t_{k})e^{\sigma_{k}(t_{k+1}-t_{k})}}$$

for neutral (*σ*_*k*_ = 0), constant (*σ*_*k*_ = *s*), and time dependent models of selection, retaining trajectories for which the constant or time-dependent models of selection outperformed the neutral model. This was evaluated using the Bayesian Information Criterion (BIC) to account for the increased complexity of the models including selection [96].

### Construction of haplotypes

For each individual, only potentially non-neutral loci were retained for the rest of the analysis, with each combination of alleles at these sites representing a haplotype. We converted the sequence data into a set describing the number of times each haplotype was observed in the sequence data, at each sampling time point. For example, if three non-neutral loci were identified, we might count the number of reads with the alleles G, A, and C at these loci at a given time point; the proportion of such reads would specify the observed frequency of the haplotype GAC. Only viral haplotypes which were observed in the sequencing data were considered, generally representing a small fraction of the haplotypes that could potentially exist. This approximation is equivalent to the assumption that non-observed viral haplotypes were under sufficiently strong purifying selection to prevent them from reaching an observable frequency.

### Multi-locus evolutionary models

Our evolutionary models considered the effect of mutation, selection, and recombination upon the population. Within a model the frequency of the haplotype *a* at generation *t*_*k*_ of the within-host viral population was specified by the frequency *q*_*a*_*(t*_*k*_*)*, frequencies changing according to the three evolutionary processes. The model system was propagated within the space of observed haplotypes using a Wright-Fisher approach of discrete generations, with successive steps of mutation, recombination and selection.

Mutation was approximated as occurring between haplotypes that differ by a single nucleotide with rate *μ* per generation. Recombination was approximated as occurring in a pairwise manner between haplotypes with rate *ρ* per base per generation. That is, if a recombination event occurring between the loci indexed *i* and *i+1*, and involving the haplotypes *a* and *b*, were to produce the haplotype *c*, then in our model the new haplotype was produced at rate *Δ*_*i*,*i+1*_*ρq*_*a*_*(t*_*k*_*)q*_*b*_*(t*_*k*_*)* where *Δ*_*i*,*i+1*_ is the sequence distance between loci *i* and *i+1*.

A time-dependent model of selection acting upon haplotypes was applied, simulating the changing selection acting upon HIV during an infection. The time-dependent fitness *w*_*a*_ of a haplotype *a* at time *t* was modelled via a hierarchical model of single-locus terms

$$w_{a}(t)=\text{exp}\left(\sum_{i}\sigma_{i}I_{\left\{i,a,T_{i}\right\}}\right)$$

where the sum is calculated over all loci i in the set of potentially non-neutral loci identified above. The parameter *s*_*i*_ denotes selection acting for or against all haplotypes with a variant at locus *i*, and the parameter *I*_*{i*,*a*,*Ti}*_ is a binary indicator function. Here, T_i_ is the time at which selection begins to act upon the variant allele at locus i. The indicator function is set so that *I*_*{i*,*a*,*Ti}*_ = 1 if t>T_i_, and if the haplotype *a* contains the variant allele at locus *i*, while *I*_*{i*,*a*,*Ti}*_ = 0 if it is true either that *a* does not contain the variant allele at i, or if *t* ≤ *T*_*i*_.

Selection then modifies the frequency of each haplotype according to the equation

$$q_{a}(t+1)=\frac{w_{a}(t)q_{a}(t)}{\sum_{b}w_{b}(t)q_{b}(t)}$$

where the sum with index *b* indicates a sum over all haplotypes, including *a*. In this manner the frequency of a haplotype changes according to the relation between its fitness and the mean fitness of the total viral population.

On the basis of previous studies[77,97–100], parameters for mutation and recombination were set at *μ* = 3 x 10^−5^ per generation and *ρ* = 10^−5^ per base per generation, with a generation time of two days[101], reflecting parameters derived from studies of chronic infection. Parameters *σ*_*i*_ and *T*_*i*_, and initial frequencies *q*^*a*^*(t*_*0*_*)*, were learnt from the data according to a hierarchical model framework. An initial, neutral model contained no selection parameters, every haplotype having equal fitness. Next, single-locus selection models were considered. Such a model uses the set of parameters i, a, *σ*_*i*_, and *T*_*i*_, to describe the locus and allele at which selection acted, the magnitude of selection and the time at which selection took effect. In each model, given the loci and allele under selection, the optimal values for the magnitudes and times of selection were identified, using a simple likelihood optimisation process. The Dirichlet multinomial likelihood described above, with the inferred noise parameter C, was used in this process, matching the observed haplotype frequencies to those produced by the evolutionary model. To improve coding efficiency a model lacking mutation and recombination was used to derive reasonable starting parameters for selection and haplotype frequencies; the subsequent application of a model with the addition of mutation, then the full model with mutation and recombination gave the final likelihood and maximum likelihood parameters. Replicate calculations with different random seeds were used to validate likelihood calculations. By means of an iterative process, more complex models of selection were considered. Initially, selection parameters were added, taking the best n-locus models and adding selection at an additional locus to each one. Upon the discovery of a model for which adding selection at a further locus did not improve the model, a process involving both the addition and subtraction of parameters was initiated, to the point of discovering a model for which neither adding a further pair of selection parameters, or removing an existing pair of parameters, improved the model. The comparison of models was performed using the Bayesian Information Criterion. As a conservative step, a model with an additional pair of selection parameters was required to significantly outperform a simpler model to be accepted, this being denoted by an improvement of 10 units of BIC given a maximum likelihood set of parameters for each model. Compared to an earlier model of selection at multiple loci [28], our approach has the advantage of parsimony, inferring selection at a locus only where there is specific evidence for non-neutrality at that site. Our model of time-dependent selection accounts for the expected behaviour of the host immune system against HIV. No prior distribution of selection coefficients was assumed.

### Estimating confidence intervals for selection coefficients

Confidence intervals were calculated for each parameter *s*_*i*_ inferred in the maximum likelihood calculation. Supposing the maximum log likelihood for a given inferred system to be equal to some value L, error bars were generated via a constrained exploration of the model space, in which a change in model parameters was accepted if the resulting likelihood was not greater than L-2, and for which changes to the parameter of concern, *σ*_*i*_, were constrained so that this parameter could only change in a specific direction; forcing this parameter to increase generated an estimate, after repeated iteration, for the upper error bar of this parameter, while forcing this parameter to decrease generated an estimate of the lower error bar of this parameter.

### Reporting selection coefficients

In our study we report the respective fitness advantage conferred by each beneficial mutation as a percentage per generation. This statistic, s, is calculated from an inferred selection coefficient as

$$s=e^{\sigma_{i}}-1$$

### Direction of selection

For each of the selected mutations identified in our analysis, we determined whether evolution was towards or away from the subtype-specific population consensus. The subtype of each gene region for each individual and the population consensus for subtypes A, D and C in Uganda during a similar period to which the individuals were sampled was previously determined[31].

### Amino Acid position association with immune escape

Using the Los Alamos HIV database (http://www.hiv.lanl.gov), we determined for each of the AA positions in the p24 and gp41 regions that we analysed whether they have previously been associated with changes in CTL susceptibility or neutralising antibodies. For CTL susceptibility we used the Epitope Variant and Escape Mutation Database CTL variant search tool (https://www.hiv.lanl.gov/content/immunology/variants/variant_search.html?db=ctl). AA positions were marked as being associated with susceptibility to CTLs if susceptible and/or resistant variants were returned in the search tool. We excluded codons inferred to be susceptible to CTLs if the only evidence was high levels of diversity at the population level. For neutralising antibodies we used the Neutralizing Antibody Contacts and Features search tool (https://www.hiv.lanl.gov/components/sequence/HIV/featuredb/search/env_ab_search_pub.comp), marking codons as associated with susceptibility if variants were shown or predicted to affect neutralisation by antibodies, or binding to neutralising antibodies.

### Codon diversity

We used the diversity statistic π to measure mean intra- and inter-host diversity at the codon level. Given a locus *a* at which the read depth is *N*, where *n*_*a*_ of each codon a was where *n*_*i*_ of each of the three-nucleotide codons *i* have been observed, we define the diversity statistic π as

$$\pi =\frac{N(N-1)-\sum_{i}n_{i}(n_{i}-1)}{N(N-1)}.$$

This diversity statistic has been shown to be less prone than some other metrics to biases at the intra-host scale[102], and using codons (nucleotide triplet motifs) rather than amino acids or single nucleotides means our measure incorporates synonymous and nonsynonymous diversity whilst still enabling comparison with information on sensitivity to host immune responses, which is typically given at the amino acid level. To determine the mean intra-host diversity at a given codon position, we calculated π for each individual and then took the mean for all 34 individuals. To determine the mean inter-host diversity at a given codon position we calculated π for each subtype and then took the mean for all three subtypes (A, C and D).

### Application of the method to simulated data

The performance of our method was evaluated using simulated data, generated in order to be as close in nature as possible to the real data. A complete discussion of the methods used is given in S1 Text.

## Supporting information

S1 Fig Codon positions containing nucleotide sites that are inferred to be under selection. This includes codons that are genuinely under selection and those that are increasing in frequency due to hitchhiking. Codon positions are in relation to the HXB2 reference sequence, and the y-axis gives the number of times that codon is inferred to be under selection across 34 individuals. Occasionally the same codon is inferred to be under selection twice in the same individual. Pink: codons associated with changes in susceptibility to CTLs; Purple: codon probably affects susceptibility to CTLs; Blue: codons associated with susceptibility to NAbs; Cyan: codons in an epitope position targeted by the neutralising antibody HGF24 in some African isolates. Where a codon is implicated in multiple responses, for clarity they are coloured in order of preference NAb, CTL, NAb (likely).
(TIF)

S2 Fig Magnitudes of selection for variants that were inferred to be under selection, or not inferred to be under selection, in simulated systems in which the full data about the evolution of the system was available to the inference code. A magnitude of 0.1 corresponds to a 10% fitness advantage per generation. More weakly selected variants were less likely to be identified as such. A variant will fail to be identified as under selection if it makes too small an impact upon the evolution of the system to be detected by our code, which adopts a parsimonious approach to identifying selected variants. Such an event can occur for a variety of reasons. For example if a newly-selected variant exists at very low frequency, and if the addition of selection for this variant is insufficient to raise the fitness of sequences carrying it to a value above the mean population fitness, selection will not impact the population in a way so as to be detectable.
(TIF)

S3 Fig Distributions of input and inferred magnitudes of selection for simulated data in which the observed data described A. the full region of the virus simulated, containing all variants under selection and B. A fraction of the simulated region of the virus. Data are shown for variants at which the magnitude of selection could be inferred with confidence.
(TIF)

S4 Fig Observed (solid lines) and inferred (dashed lines) haplotype frequencies for simulated data in which all loci under selection were observed. In some cases the lines cannot be distinguished from one another.
(TIF)

S5 Fig True and inferred magnitudes and timings of selection for simulated data. Confidence intervals for the inferred selection coefficients are shown, calculated using the method described in the main text. The red dashed line indicates agreement between the true and inferred parameters. We note that in some cases, confidence intervals for selection coefficients are large, as was the case for our inferences from the biological data. This can occur, for example, where data is not collected at sufficient time resolution to quantify selection; for a sudden fixation event only a lower bound for selection can clearly be identified.
(TIF)

S6 Fig Observed (solid lines) and inferred (dashed lines) haplotype frequencies for simulated data in which only data from within a fraction of a simulated region was observed. In some cases the lines cannot be distinguished from one another.
(TIF)

S7 Fig No correlation between time of onset and strength of selection. Linear regression, p24, p = 0.20; gp41, p = 0.83.
(TIF)

S8 Fig Proportion of mutations inferred to be under selection that are towards population level consenus or are nonsynonymous. This includes codons that are genuinely under selection and those that are increasing in frequency due to hitchhiking. In all cases mutations are grouped according to the number of times the codon in which they appear is inferred to be under selection across the 34 individuals (x-axis). Top row: the number of codons in each group. Middle row: the proportion of mutations in each group that are towards population level consensus. Bottom row: the proportion of mutations in each group that are nonsynonymous.
(TIF)

S9 Fig Illustration of the construction of haplotypes. Using sequence data from a single region in a single patient, loci containing potentially non-neutral trajectories were identified. Alleles present at these loci were combined to construct haplotypes. The number of observations of each haplotype in the sequence data was counted for each time point at which the population was sampled. Inferences were performed using these haplotype counts.
(TIF)

S10 Fig Sets of nucleotide trajectories that were identified as putatively hitchhiking. These trajectories were used to create a conservative estimate of the extent of noise in the sequencing data.
(TIF)

S1 Table Summary of results for all sites inferred to be under selection (XLSX)

S2 Table Characteristics of all codons analysed: Sensitivity to host immunity, within- and between-host diversity, and the number of times the codon was inferred under selection (XLSX)

S3 Table True and inferred selection parameters for a case in which sequence data describes all selected alleles within a system. (XLSX)

S4 Table True and inferred selection parameters and times for a case in which sequence data partially describes the selected alleles within a system. We here simulated the evolution of populations of viruses, each comprised of genotypes containing 24 polymorphic alleles (numbered 1 to 24 for convenience; genotype positions are also provided). The columns showing the true model parameters describe, for each of the 20 simulated systems, which loci were modelled as being under selection (seven loci were chosen at random in each case, the remainder being neutral), the time at which selection for the variant at each locus began, and the magnitude of selection acting on this variant. The columns showing inferences show the loci at which selection was inferred to act, and for each of these loci the time at which selection was inferred to begin, and the magnitude of selection inferred to act upon the variant allele. Inferences were conducted using a partial set of data restricted to a description of changes in the system occurring between loci 441 and 759 in the genotype making it impossible to infer selection acting at loci outside of this window.
(XLSX)

S1 Text Calculations performed on simulated data, where targeted reads describe the complete evolution of the system, and where targeted reads do not describe the complete evolution of the system. (DOCX)
