# Supplementary figures and images for: A de novo approach to inferring within-host fitness effects during untreated HIV-1 infection
Source: PLoS Pathog. 2020 Jun 3;16(6):e1008171. doi: 10.1371/journal.ppat.1008171 (PMC7295245; doi:10.1371/journal.ppat.1008171)

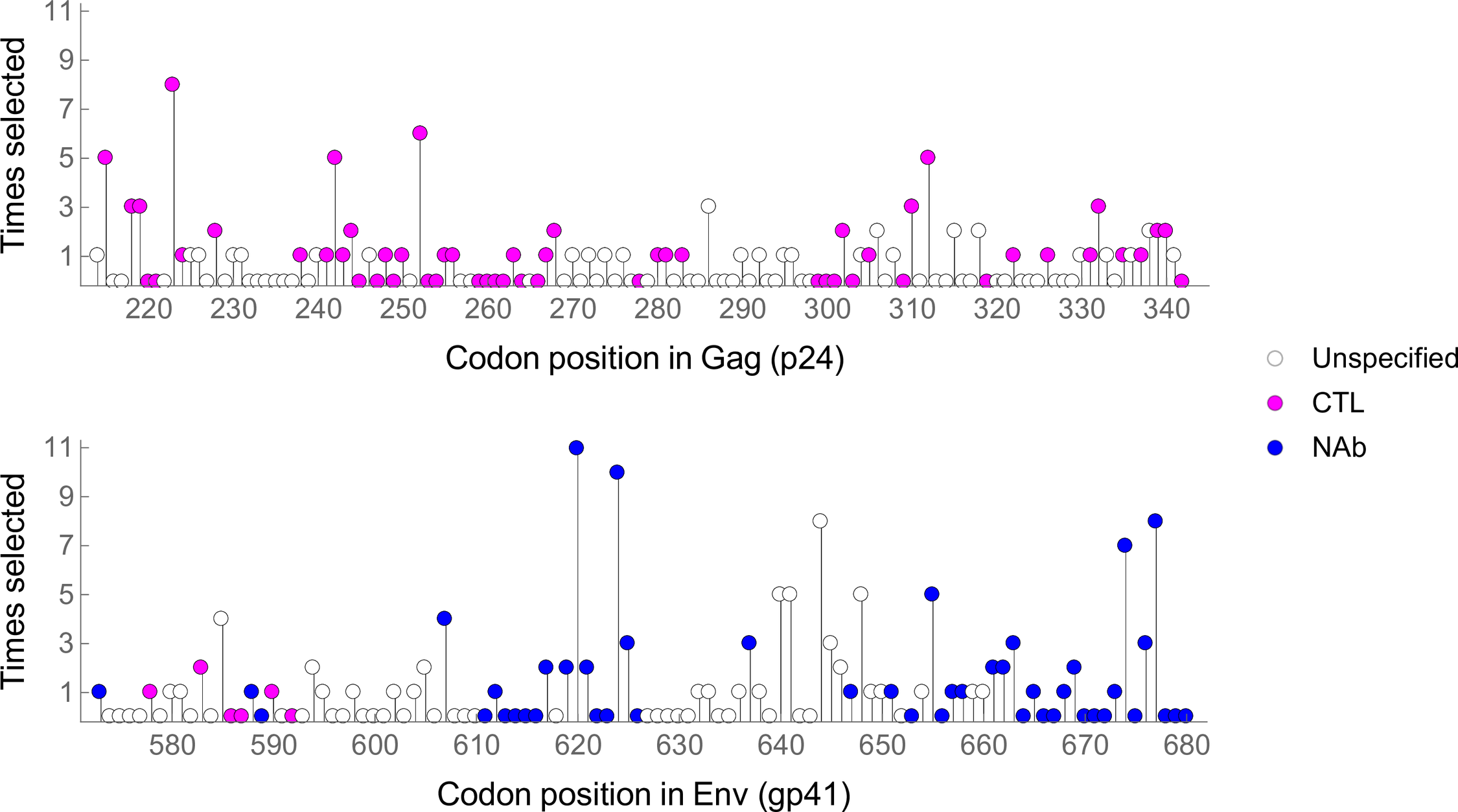

Supplement: S1 Fig — This includes codons that are genuinely under selection and those that are increasing in frequency due to hitchhiking. Codon positions are in relation to the HXB2 reference sequence, and the y-axis gives the number of times that codon is inferred to be under selection across 34 individuals. Occasionally the same codon is inferred to be under selection twice in the same individual. Pink: codons associated with changes in susceptibility to CTLs; Purple: codon probably affects susceptibility to CTLs; Blue: codons associated with susceptibility to NAbs; Cyan: codons in an epitope position targeted by the neutralising antibody HGF24 in some African isolates. Where a codon is implicated in multiple responses, for clarity they are coloured in order of preference NAb, CTL, NAb (likely). (TIF) [file ppat.1008171.s001.tif]

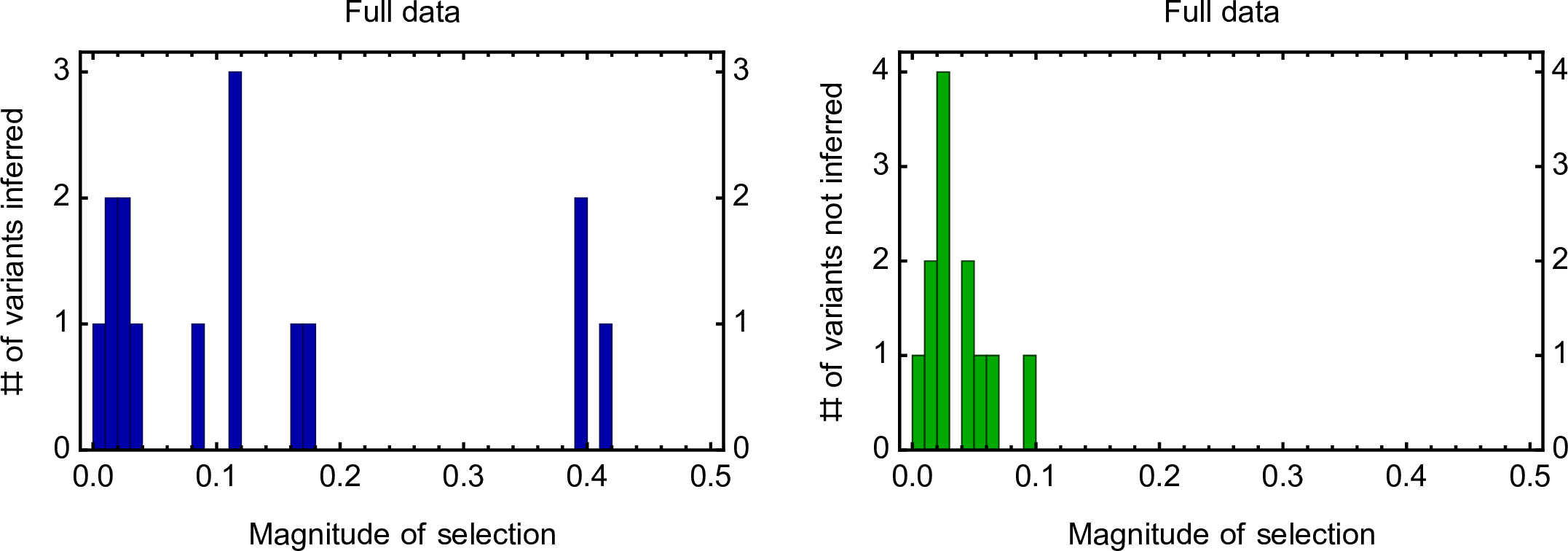

Supplement: S2 Fig — A magnitude of 0.1 corresponds to a 10% fitness advantage per generation. More weakly selected variants were less likely to be identified as such. A variant will fail to be identified as under selection if it makes too small an impact upon the evolution of the system to be detected by our code, which adopts a parsimonious approach to identifying selected variants. Such an event can occur for a variety of reasons. For example if a newly-selected variant exists at very low frequency, and if the addition of selection for this variant is insufficient to raise the fitness of sequences carrying it to a value above the mean population fitness, selection will not impact the population in a way so as to be detectable. (TIF) [file ppat.1008171.s002.tif]

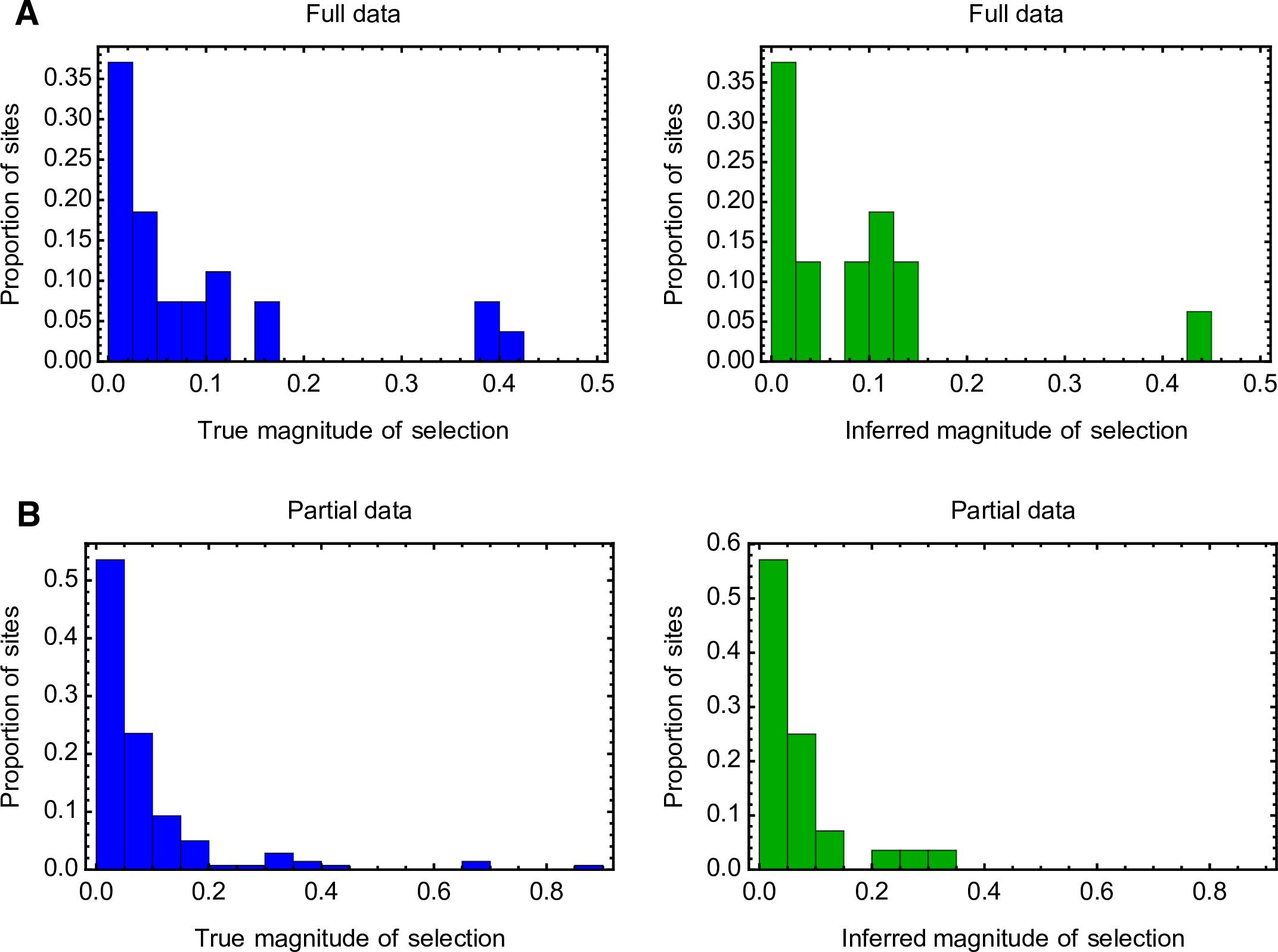

Supplement: S3 Fig — Distributions of input and inferred magnitudes of selection for simulated data in which the observed data described A. the full region of the virus simulated, containing all variants under selection and B. A fraction of the simulated region of the virus. Data are shown for variants at which the magnitude of selection could be inferred with confidence. (TIF) [file ppat.1008171.s003.tif]

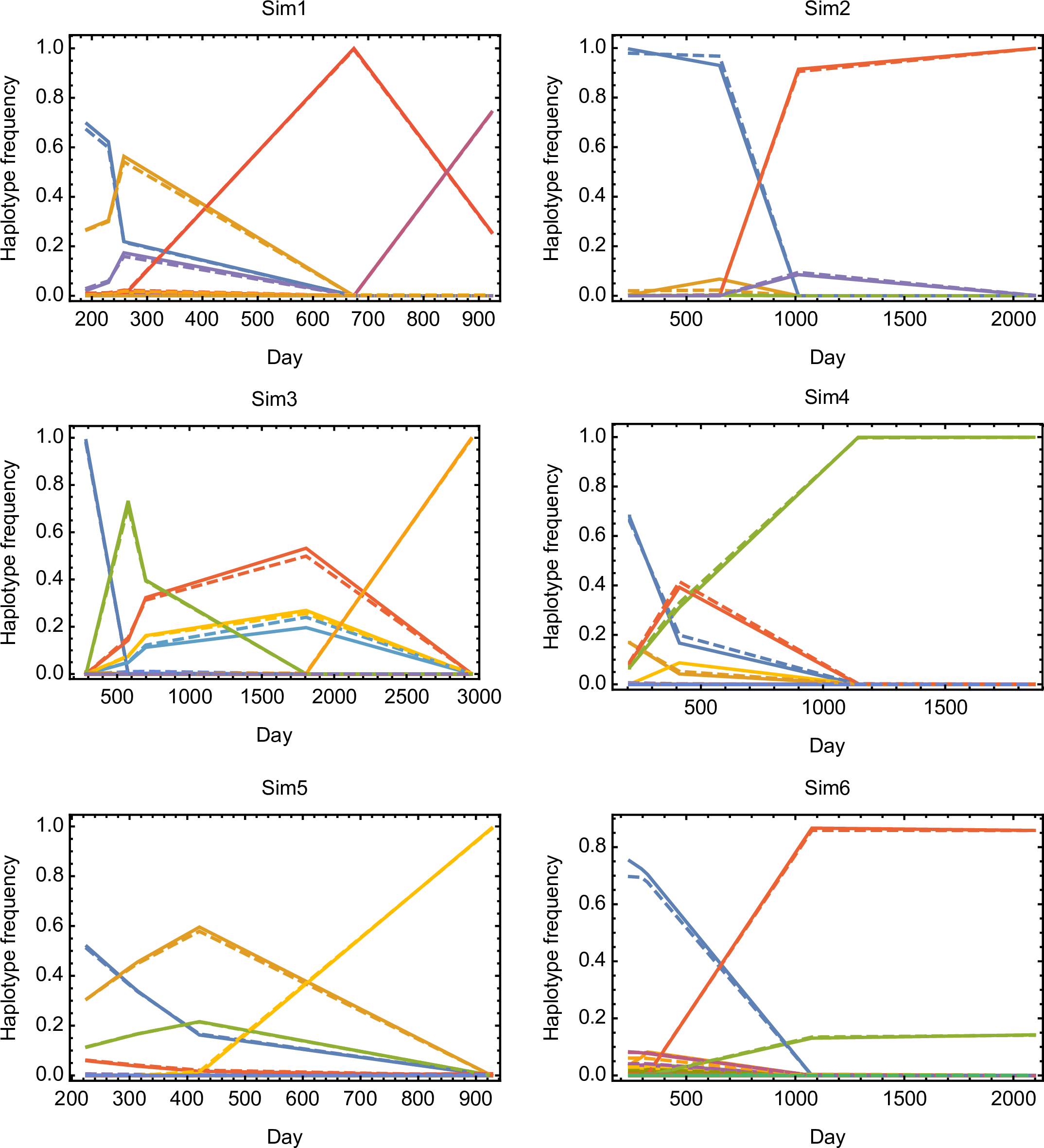

Supplement: S4 Fig — In some cases the lines cannot be distinguished from one another. (TIF) [file ppat.1008171.s004.tif]

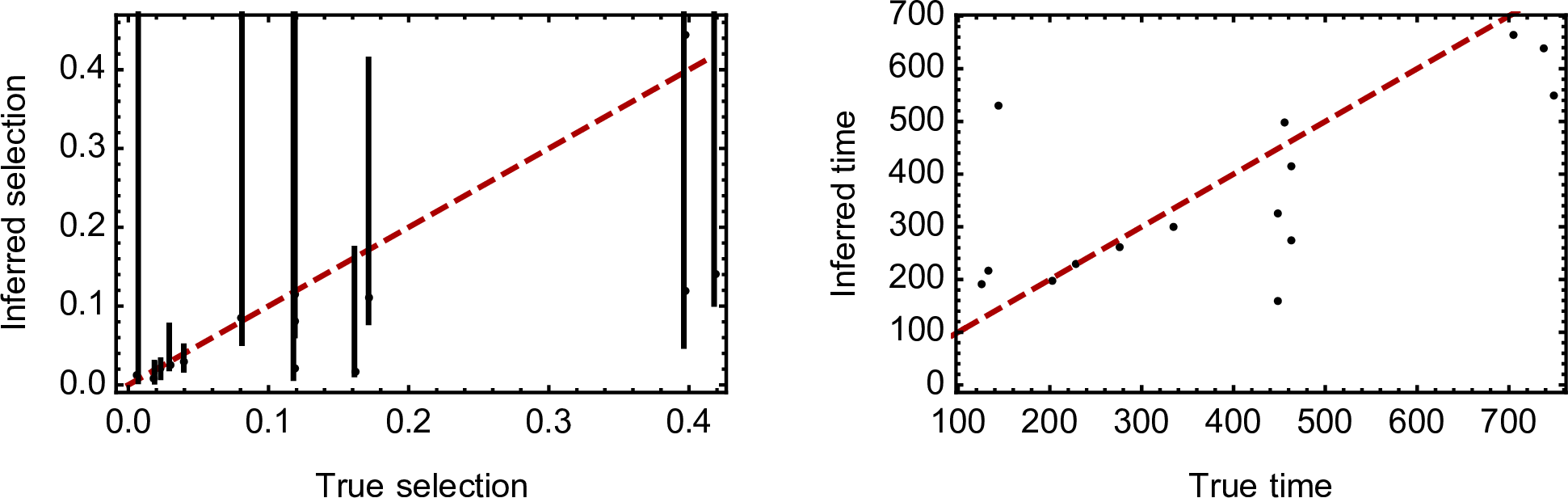

Supplement: S5 Fig — Confidence intervals for the inferred selection coefficients are shown, calculated using the method described in the main text. The red dashed line indicates agreement between the true and inferred parameters. We note that in some cases, confidence intervals for selection coefficients are large, as was the case for our inferences from the biological data. This can occur, for example, where data is not collected at sufficient time resolution to quantify selection; for a sudden fixation event only a lower bound for selection can clearly be identified. (TIF) [file ppat.1008171.s005.tif]

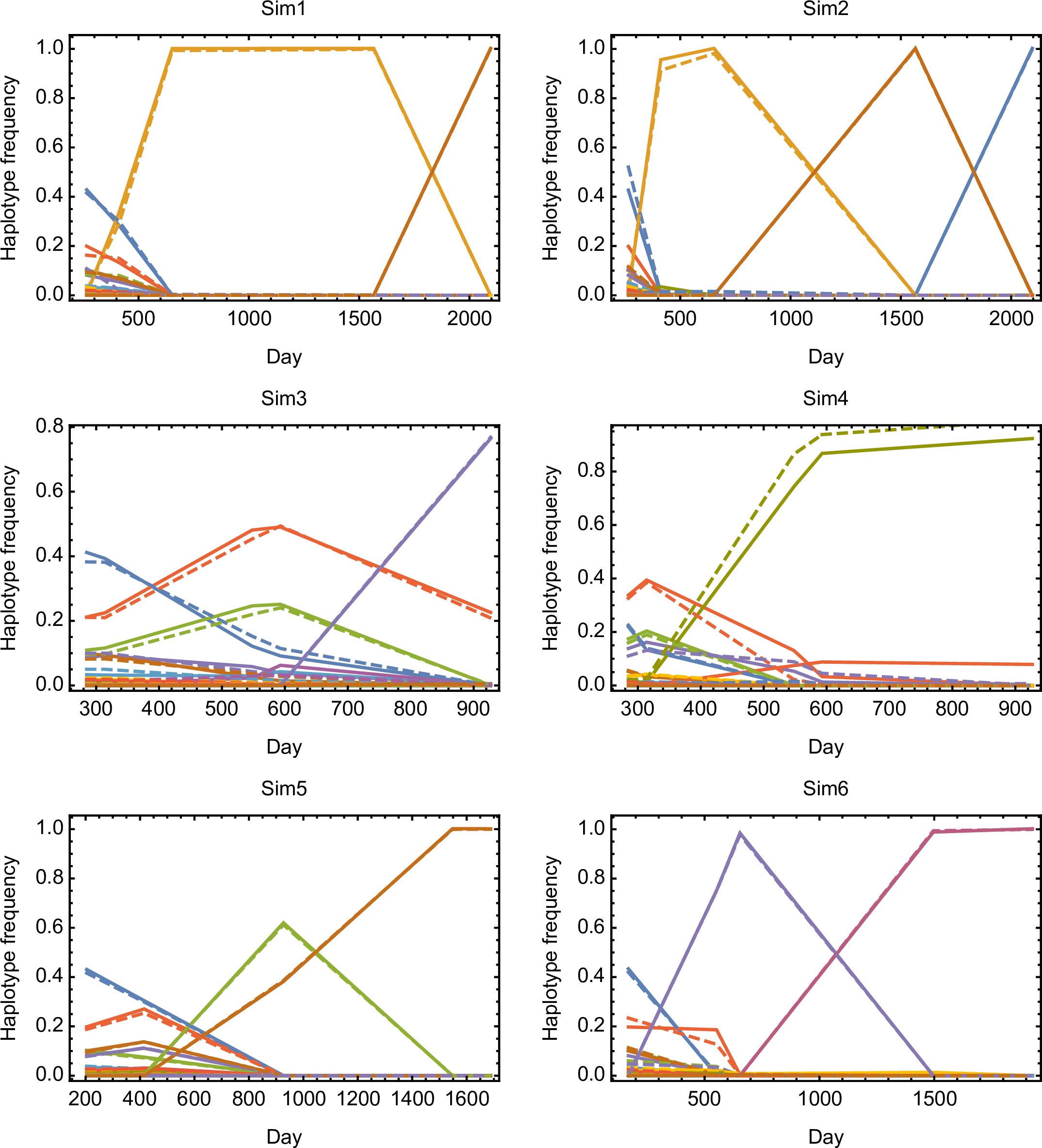

Supplement: S6 Fig — In some cases the lines cannot be distinguished from one another. (TIF) [file ppat.1008171.s006.tif]

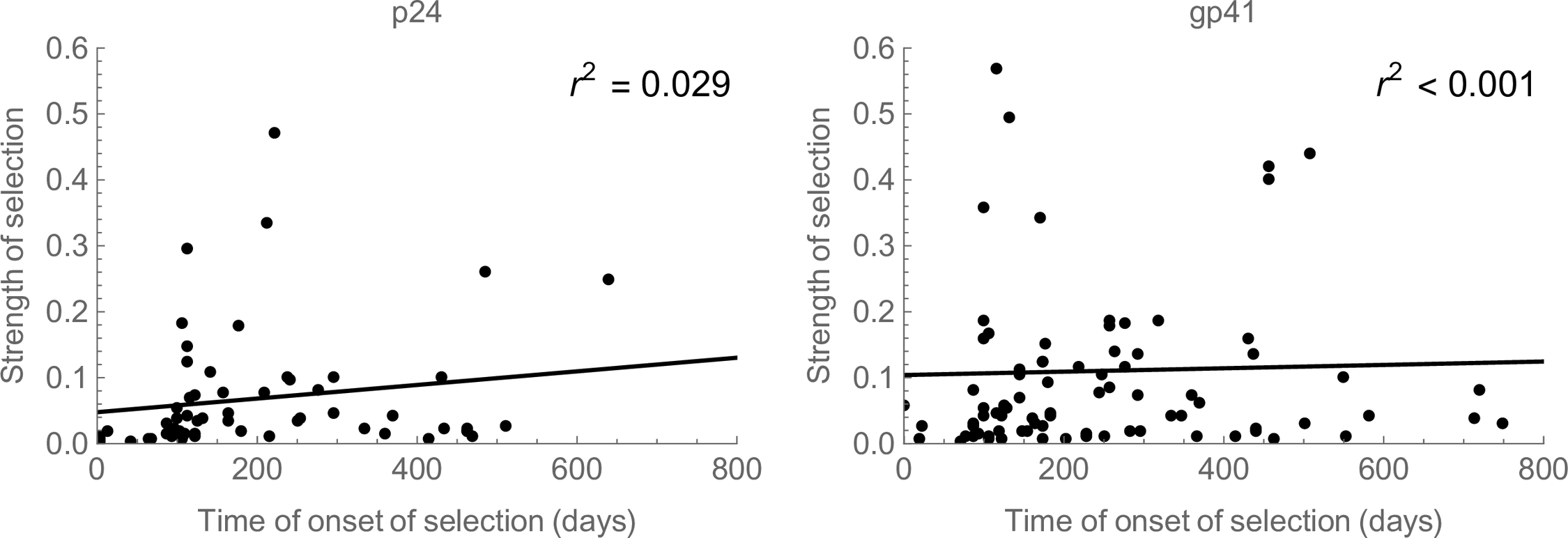

Supplement: S7 Fig — Linear regression, p24, p = 0.20; gp41, p = 0.83. (TIF) [file ppat.1008171.s007.tif]

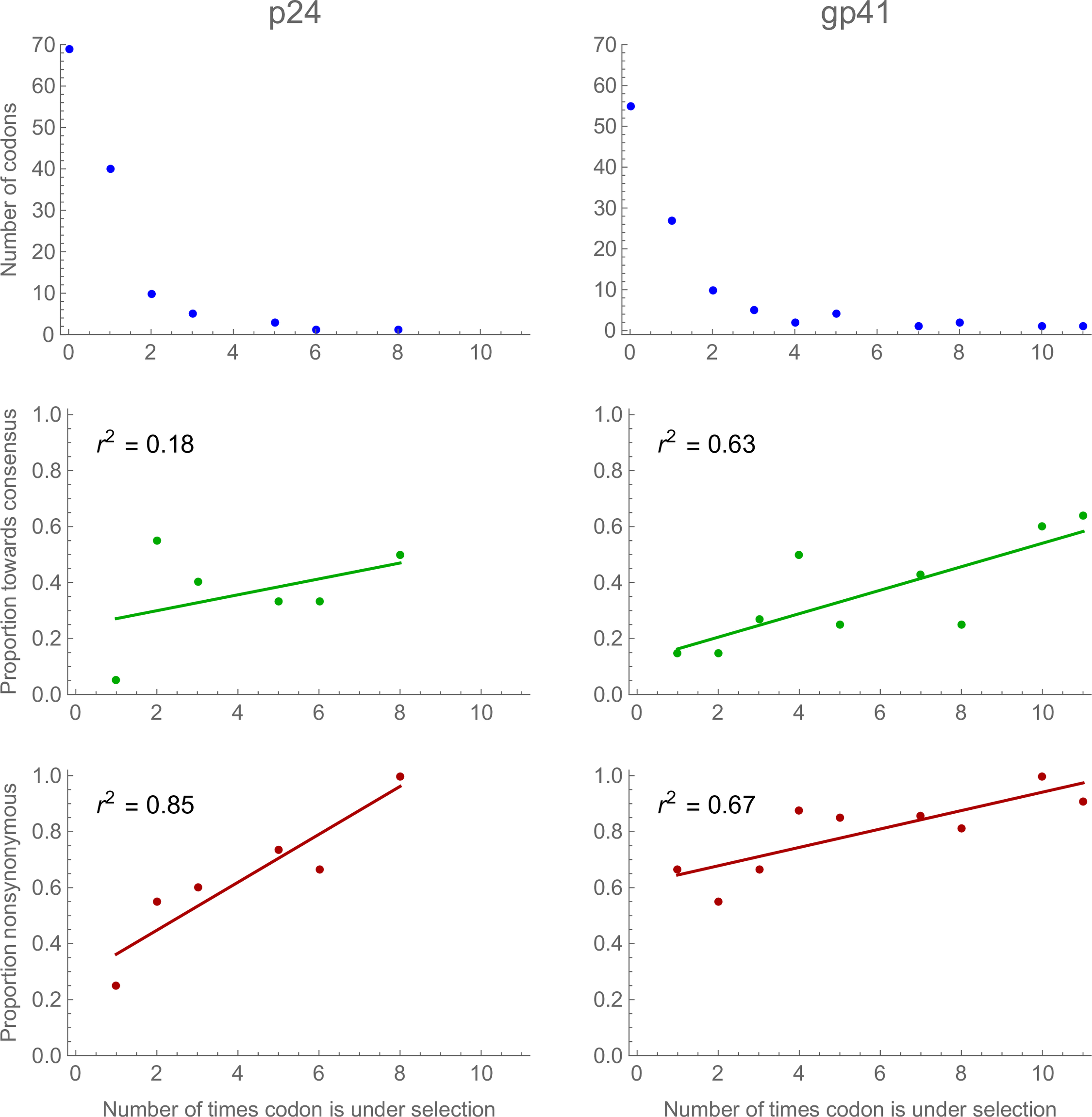

Supplement: S8 Fig — This includes codons that are genuinely under selection and those that are increasing in frequency due to hitchhiking. In all cases mutations are grouped according to the number of times the codon in which they appear is inferred to be under selection across the 34 individuals (x-axis). Top row: the number of codons in each group. Middle row: the proportion of mutations in each group that are towards population level consensus. Bottom row: the proportion of mutations in each group that are nonsynonymous. (TIF) [file ppat.1008171.s008.tif]

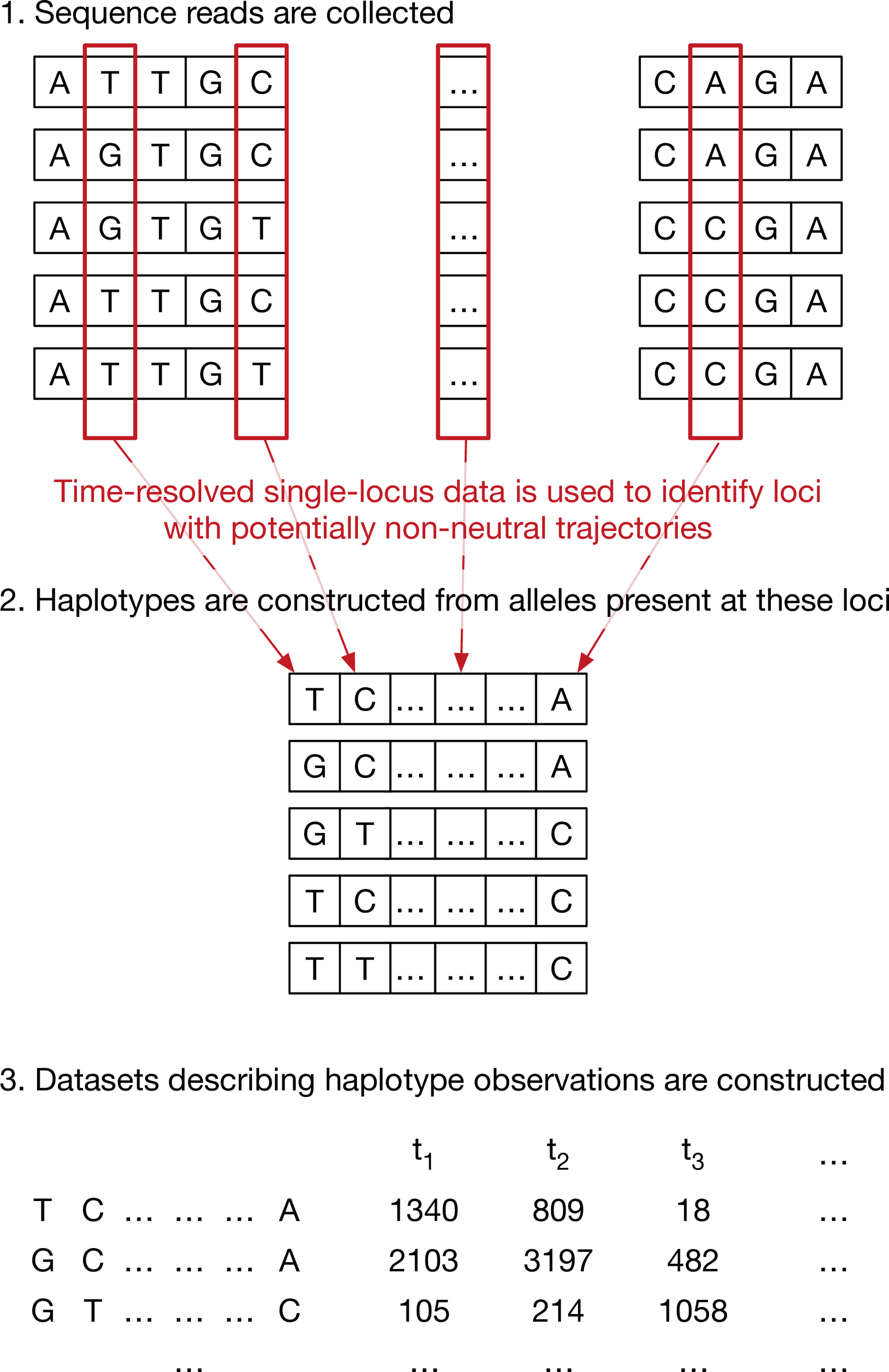

Supplement: S9 Fig — Using sequence data from a single region in a single patient, loci containing potentially non-neutral trajectories were identified. Alleles present at these loci were combined to construct haplotypes. The number of observations of each haplotype in the sequence data was counted for each time point at which the population was sampled. Inferences were performed using these haplotype counts. (TIF) [file ppat.1008171.s009.tif]

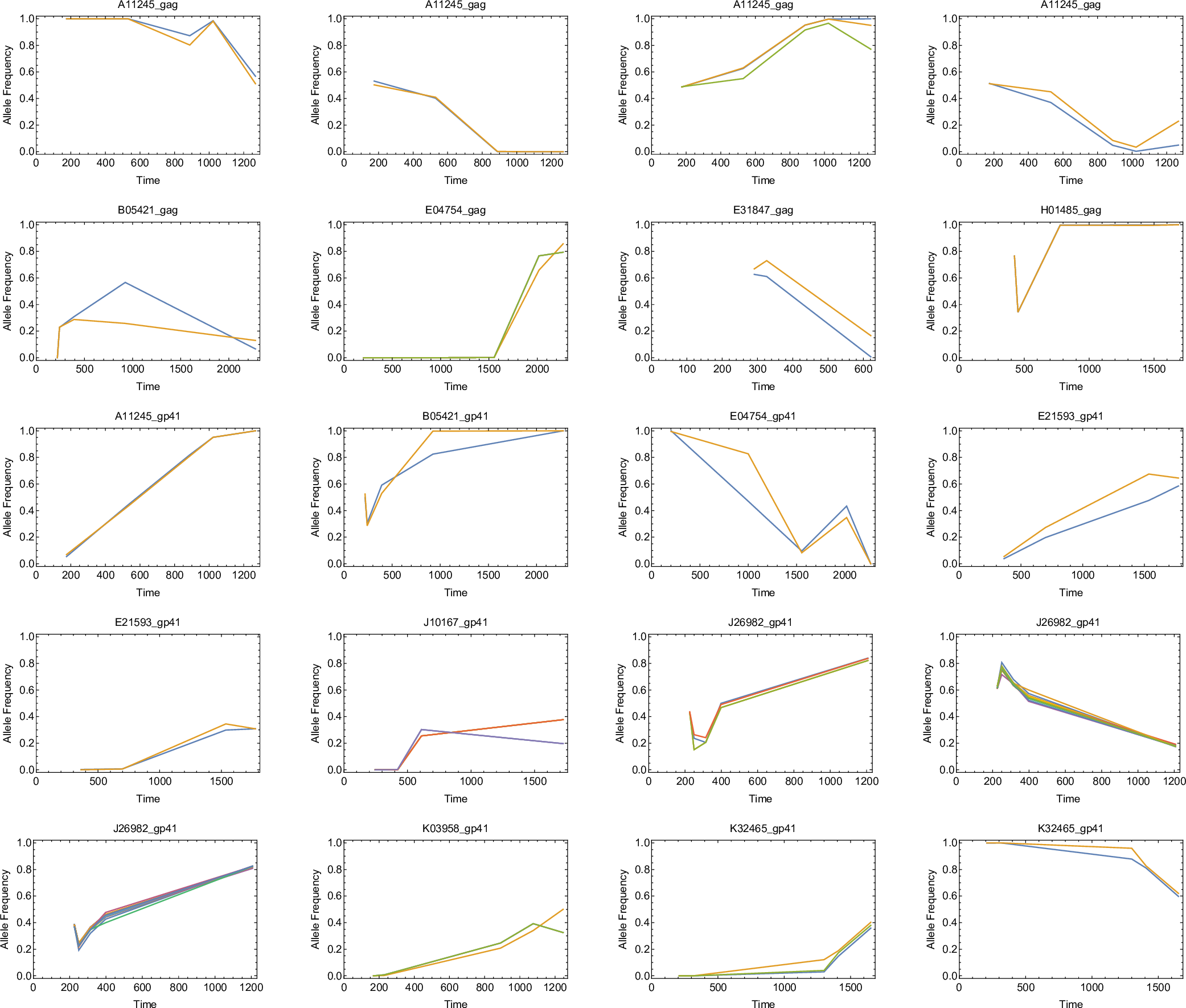

Supplement: S10 Fig — These trajectories were used to create a conservative estimate of the extent of noise in the sequencing data. (TIF) [file ppat.1008171.s010.tif]
