# Supplementary material for: A de novo approach to inferring within-host fitness effects during untreated HIV-1 infection
Source: PLoS Pathog. 2020 Jun 3;16(6):e1008171. doi: 10.1371/journal.ppat.1008171 (PMC7295245; doi:10.1371/journal.ppat.1008171)
Supplement: S1 Text — (DOCX) [file ppat.1008171.s015.docx]

**Calculations performed on simulated data**

*Inference from simulated data*

We conducted simulations to evaluate the performance of our inference method.

A key issue with the data, noted in the main text, is that the targeted sequence data we analyse has the potential to miss selected alleles which are in linkage disequilibrium with variants that we observe. Unobserved variants cannot by any method be inferred to be under selection. Our inference code was therefore applied to simulated HIV sequence data generated under two sets of circumstances.

Firstly, in order to test the inherent ability of the code to infer correct evolutionary parameters, data was generated from simulated HIV sequence data in such a way that the data described all of the alleles in the simulated system that evolved under selection. Inferences from these data illustrate the intrinsic performance of the method in identifying variants under selection, and characterising the strength of selection acting upon them.

Secondly, data were generated from simulated data in such a way that sequence reads covered only one third of the simulated region. In this case, some sites that evolved under selection were not observed, making the correct inference of selection at these sites impossible. In such a case, the potential exists for false inferences of selection to be generated, for example assigning selection to alleles that evolve in linkage disequilibrium with unobserved selected sites. Here, a key question was the estimation of the rate at which these false positive inferences occur; we also compared the magnitude of selection coefficients inferred to act upon variants (whether or not selection was correctly attributed to variants) to the ‘true’ distribution of selection coefficients input into the system.

In performing simulations, our interest was in generating data that is as close as possible to a real case of HIV evolution, in terms of the frequencies of haplotypes that might be observed in a population, the timing, and the magnitude of selection as it acts upon the population. In so far as our best estimates of ‘real’ behaviour come from inferences we have performed on the real data, we fed our inferred parameters back into a simulation, using these parameters to generate simulated data. In each simulation the haplotypes (namely the sets of alleles observed at potentially non-neutral loci) and inferred initial haplotype frequencies from one of the real datasets was used to specify the initial state of the population. Selection coefficients and times at which selection began to act upon an allele, were chosen at random from the set of inferred parameters derived from the real data. Times at which the population was observed were chosen at random from the real observation times, with the constraint that the last observation time had to be after the last time at which selection began to act upon an allele, avoiding cases in which it would be impossible to infer the presence of selection for the given allele. The depth of sampling was chosen to be the mean read depth from our real data, equal to 8195; this read depth was used for all samples.

The parameters thus chosen were used to generate a forward simulation of the system, using the biological mutation and recombination rates described in the main text.

The steps we take to make our simulated data approximate a realistic HIV population do not influence the output of our inference code. The inference process, applied to the simulated data, may in theory reproduce or fail to reproduce the parameters underlying the input data. However, the steps we take in this respect ensure that the performance of our code was assessed against data that were as close as possible to a realistic system.

**Note on the identification of selected alleles**

Previous studies involving the use of simulated data have highlighted challenges in the association of selection with specific alleles given data from an evolving system [[1]](https://paperpile.com/c/eXmZGR/zmNq).  In some cases, while the fitness of distinct haplotypes can be inferred, the attribution of selection to a specific allele is in some cases not mathematically possible.  For example, if a system consists of only two haplotypes, with the nucleotides AA and TT, and it is inferred that TT is beneficial relative to AA, it is not possible to identify the locus, or loci, at which selection acts; while the fact of selection might be inferred, the correct assignment of selection to a specific locus is not achievable.

A further issue arises with time-dependent selection.  Although in choosing parameters we aim to replicate the kind of system inferred to exist in the real data, the randomly chosen parameters may lead to cases where the effect of selection cannot be observed from the system.  For example, we may consider a population with the genotypes AT and GC. Here, if the A to G variant is under positive selection, the GC genotype will increase in frequency at the expense of the AT genotype. If at some subsequent point weaker selection against the T to C variant commences, the relative fitness of GC will be decreased.  However, the detection of the second selected variant is only possible if the AT haplotype exists at an appreciable frequency in the population at the time selection commences. If the GC variant fixes before the onset of selection against the T allele, the change in haplotype fitness will not be possible to infer from the sequence data.

**Results from Case 1: Reads describe the complete evolution of the system**

Our method performed well in identifying selected alleles where the complete evolution of the system was observed. Despite a general under-calling of variants under selection, there was a low rate of false positive calls of selection. In each simulation, a random real system was chosen, using the haplotypes and initial frequencies inferred for this system, were used to specify the initial state of the simulated system.

A set of five distinct polymorphic loci were randomly chosen, with selection being modelled to act upon the minority allele (according to the initial composition of the population) at each of these loci.  Variant alleles at other loci were modelled as not being subject to direct selection. We intended to generate five simulated populations in this manner, however for one of the chosen systems only three loci were present in the haplotypes, limiting the number of loci that could be chosen to be under selection.  We therefore conducted a sixth simulation, giving five simulations of five selected loci and one of three selected loci. Data were collected from the system at five distinct random times, in the manner described above.

Application of our inference method showed a general under-calling of variants under selection.  Of 28 selected variants in the six systems, a total of 15 were correctly inferred to be under selection. One false positive inference was made of a variant not under selection.  The results of these simulations are given in S3 Table.

Examination of haplotypes frequencies from the simulation and from the best inferred model showed a close agreement between the pattern of haplotype frequencies over time (AS4 Fig).  This behaviour suggests that the observed under-calling of sites under selection likely reflects a pattern of unobservable selection, whereby selection acting for certain alleles has little or no effect on the observable system.  Our simulations show that, given data that describes the complete range of alleles at which selection acts during the course of an infection, our method performs well. The inference process may miss a number of true variants that are under selection, and may include occasional false positive calls of selection, but where we correctly identify selection acting on a selected variant, our inferred confidence interval is likely to contain the true magnitude of selection.

In the case for which all alleles under selection were observed, the inferred selection parameters were in generally good agreement with the input values. Where a variant was correctly inferred to be under selection, the true magnitude of selection acting upon the variant was always within the confidence interval described by our likelihood-based method (S5 Fig).  However, the maximum likelihood of the inferred values was generally lower than that of the corresponding real parameter; the mean inferred selection coefficient was 8.7%, compared to a mean of 14.1% advantages for variants at which selection was inferred; this result may indicate a tendency to underestimate large selection coefficients, which may be poorly characterised due to the limited resolution of time-resolved data.

An examination of the selection coefficients of identified and non-identified selected variants showed that variants under stronger magnitudes of selection were more likely to be identified as such (AS2 Fig). At low magnitudes of selection the effect of selection on a variant is more likely to have a negligible effect on the viral population, preventing the inference of such variants under our approach, which maximises parsimony. The effects of underestimating higher magnitudes of selection while failing to infer weak selection combined to produce a distribution of inferred selection coefficients that was close to the total original distribution of selective effects; the distributions of inferred and real coefficients were not statistically distinguishable (p=0.982, Kolmogorov-Smirnov test; p=0.950, Mann Whitney test). The respective distributions are shown in S3 Fig A.

Timings at which selection was inferred showed a broad pattern of agreement with the correct time, albeit with some outliers (S5 Fig).

**Results from Case 2: Reads partially describe the evolution of the system**

Analysis of HIV populations has identified rapid recombination, albeit that variant sites fewer than 100 nucleotides apart do not evolve independently [[2]](https://paperpile.com/c/eXmZGR/mOBu).  Given targeted sequence reads we therefore cannot rule out the possibility that selected alleles not within the region of sequencing might, via linkage disequilibrium, cause changes in the genetic composition of the observed population; the influence of such alleles is likely to lead to errors in the inference of selection from the observed sequence data [[3]](https://paperpile.com/c/eXmZGR/2jWO).

Simulations of an extended viral genotype were conducted in order to estimate a false positive rate of inference of selection for each genetic region of HIV.  We note that the inference of selection in our approach involves i) the identification of a genetic variant as being polymorphic; ii) the inference under a single-locus model that the variant changes in frequency in a way consistent with a non-neutral model of adaptation; iii) the inference of selection acting upon a variant using a multi-locus model of evolution.  Failure at any of these three stages prevents an allele from being inferred to evolve under selection.

To investigate the inference of selection in a multi-locus system we conducted simulations of multi-locus systems spanning three times the length of the observed sequencing window.  Haplotypes and initial haplotype frequencies were generated from those inferred for the gp41 region of patient 10 and the gag region of patient 34, each of which described haplotypes with at least 24 variant alleles.  Forward simulations were conducted as for the simulations above, with the exception that the biological recombination rate was multiplied by three, simulating a region of genome three times longer than the length of the targeted sequencing region, while seven, instead of five, alleles were chosen to be under selection.  Data from the simulation was collected from a region of the simulated system of length equal to that of the original targeted sequencing, spanning 11 of the variant alleles in the system. Ten simulations were conducted for each of the two patient datasets. The results of these simulations are given in S4 Table.

Data across the 20 simulations showed a high level of false positive inferences of selection.  Fits to the data often produced a good qualitative match between observed and inferred variant frequencies (AS6 Fig); an inference of selection which closely approximated the data was not a guarantee that the result was correct.  Of a total of 56 sites inferred to be under selection, 21 were true positives, while 35 were false positives, giving a false positive rate of inference of 62.5%.  This result highlights the inadequacy of single-locus methods for inferring selection in the presence of linkage disequilibrium; even where linkage disequilibrium between a subset of variants is fully accounted for, a high false positive rate of inferring selection can be obtained.

The above false positive rate was combined with an estimate of the probability of a site being chosen for the multi-locus inference procedure to derive an overall false positive rate for our approach as a whole.   For each region of the genome, the respective rates at which synonymous and non-synonymous variants were identified as being under selection were calculated, dividing the number of identified variants by the mutational opportunity for synonymous or non-synonymous variation.  To calculate this latter statistic, we evaluated the effect of each of the three possible nucleotide substitutions at each position in the region of interest, relative to the patient-specific consensus sequence. Each nucleotide was then assigned the value 0, ⅓, ⅔, or 1 according to the proportion of substitutions that led to synonymous or non-synonymous variation; the mutational opportunities were calculated by summing these statistics across all nucleotides and all individuals.  Of the sites identified using single-locus inference methods, a variable proportion were then inferred to be under selection using our multi-locus approach; taking a deliberately conservative measure, we assumed that all such sites were inferred to be under selection. The false positive rates *p_n_* and *p_s_* of synonymous and non-synonymous mutations were then calculated; we obtained rates of *p_n_*=0.71% and *p_s_*=5.12% for variants in gag, and *p_n_*=2.28% and *p_s_*=5.03% for variants in gp41.

Taking an assumption that synonymous and non-synonymous variants are independent, the probability that across a set of *N*=34 infections *n* non-synonymous and *s* synonymous variants will falsely be inferred is given by

$$\sum_{i=n}^{\infty} \sum_{j=s}^{\infty} \left( \begin{matrix} N \\ i \end{matrix} \right)\left( \begin{matrix} N \\ j \end{matrix} \right)p_{n}^{i}{(1-p_{n})}^{N-i}p_{s}^{j}{(1-p_{s})}^{N-j}$$

Nucleotides at which variants were inferred to be under selection for that this statistic to be less than 0.05 were called, at which it was possible to say with 95% certainty, under the assumptions given, that at least one of these nucleotides was genuinely under selection.  Our approach identifies, under conservative statistical assumptions, sites in the genome at which selection shaped the evolution of the viral population.

In this case the distribution of inferred selection coefficients was again compared to the distribution of selection coefficients used in the simulations; visual inspection suggested the distributed magnitudes of selection to be similar (S3 Fig B). As detailed in the main text these two distributions could not be statistically distinguished from one another via a Kolmogorov-Smirnov test (p=0.078), though the fit was not as close as for the previous set of simulations. A Mann-Whitney test did not find a statistically significant difference between the median values of the datasets (p=0.754). While the means of the two distributions were similar, at 0.088 and 0.071 for the real and inferred coefficients respectively, the real data had a longer tail, indicated by variances of 0.0166 and 0.0058 for the two distributions.

1. [Illingworth CJR. Fitness Inference from Short-Read Data: Within-Host Evolution of a Reassortant H5N1 Influenza Virus. Mol Biol Evol. 2015;32: 3012–3026.](http://paperpile.com/b/eXmZGR/zmNq)

2. [Zanini F, Brodin J, Thebo L, Lanz C, Bratt G, Albert J, et al. Population genomics of intrapatient HIV-1 evolution. Elife. 2015;4. doi:](http://paperpile.com/b/eXmZGR/mOBu)[10.7554/eLife.11282](http://dx.doi.org/10.7554/eLife.11282)

3. [Illingworth CJR, Mustonen V. Distinguishing driver and passenger mutations in an evolutionary history categorized by interference. Genetics. 2011;189: 989–1000.](http://paperpile.com/b/eXmZGR/2jWO)
